# Downregulation of the Polycomb-Associated Methyltransferase Ezh2 during Maturation of Hippocampal Neurons Is Mediated by MicroRNAs Let-7 and miR-124

**DOI:** 10.3390/ijms21228472

**Published:** 2020-11-11

**Authors:** Laura Guajardo, Rodrigo Aguilar, Fernando J. Bustos, Gino Nardocci, Rodrigo A. Gutiérrez, Brigitte van Zundert, Martin Montecino

**Affiliations:** 1 Institute of Biomedical Sciences, Faculty of Medicine and Faculty of Life Sciences, Universidad Andres Bello, Santiago 8370186, Chile; lau.guajardoc@gmail.com (L.G.); rodrigo.aguilar@unab.cl (R.A.); fernando.bustos@unab.cl (F.J.B.); gnardocci@uandes.cl (G.N.); 2 FONDAP Center for Genome Regulation, Santiago 8370186, Chile; rgutierrez@bio.puc.cl; 3 CARE Biomedical Research Center, Santiago 83370186, Chile; 4 Millennium Institute for Integrative Biology, Departamento de Genética Molecular y Microbiología, Facultad de Ciencias Biológicas, Pontificia Universidad Católica de Chile, Santiago 8331150, Chile

**Keywords:** Ezh2 expression, microRNA-mediated control, let-7 and mir-124 in hippocampus

## Abstract

Ezh2 is a catalytic subunit of the polycomb repressive complex 2 (PRC2) which mediates epigenetic gene silencing through depositing the mark histone H3 lysine 27 trimethylation (H3K27me3) at target genomic sequences. Previous studies have demonstrated that Enhancer of Zeste Homolog 2 (Ezh2) was differentially expressed during maturation of hippocampal neurons; in immature neurons, Ezh2 was abundantly expressed, whereas in mature neurons the expression Ezh2 was significantly reduced. Here, we report that Ezh2 is downregulated by microRNAs (miRs) that are expressed during the hippocampal maturation process. We show that, in mature hippocampal neurons, lethal-7 (let-7) and microRNA-124 (miR-124) are robustly expressed and can target cognate motifs at the 3′-UTR of the Ezh2 gene sequence to downregulate Ezh2 expression. Together, these data demonstrate that the PRC2 repressive activity during hippocampal maturation is controlled through a post-transcriptional mechanism that mediates Ezh2 downregulation in mature neurons.

## 1. Introduction

Several reports have demonstrated the critical role of epigenetic mechanisms during control of gene expression associated with the physiological function of the central nervous system (CNS) in mammals [1,2]. These mechanisms include DNA methylation, histone post-translational modifications, and long and short non-coding RNA activities, among others [3]. A relevant component of epigenetic control in the CNS is mediated by the polycomb repressive complex 2 (PRC2), which catalyzes the deposit of the repressive histone mark trimethyl lysine 27 of histone H3 (H3K27me3) at target gene promoters, thereby producing their transcriptional silencing [4,5]. Our team showed that, during early stages of hippocampal neuron maturation, PRC2 activity was critical to control the expression of non-neuronal gene programs (e.g., osteogenic lineage genes), as well as the expression of key components of the dendritic arbor formation that were required at more advanced stages of maturation [6,7]. These data demonstrated that the catalytic subunit of PRC2, the methyltransferase Enhancer of Zeste Homolog 2 (Ezh2), played a key role during this gene expression silencing events in the hippocampus. Additionally, Ezh2 has been found expressed in neuronal progenitor cells and in neurons during early embryonic stages, where Ezh2 trimethylates H3K27 target regulatory sequences for controlling the balance between self-renewal and lineage commitment [8,9].

It has been determined that, in rat hippocampal neurons, the expression of Ezh2 is downregulated during development [7]. Thus, Ezh2 is significantly expressed in the hippocampus at embryonic and early postnatal stages, where, as part of PRC2, it binds to target promoters to mediate gene silencing. At later adult stages, Ezh2 mRNA and protein are largely undetectable in hippocampal neurons and Ezh2 function in the PRC2 complex can be partially replaced by Ezh1 [6,7]. Although our results indicated that an important component mediating this Ezh2 downregulation during hippocampal maturation could involve transcriptional inhibition of the Ezh2 gene, additional evidence has demonstrated the contribution of post-transcriptional and post-translational mechanisms, including the role of microRNAs [10] and ubiquitin-dependent proteasome degradation [11].

Accumulating evidence indicates that microRNAs (miRs) are key modulators of gene expression in the CNS [12,13], where approximately 50% of the total known mammalian miRs are expressed [14,15], regulating critical processes that include dendritogenesis [16] and synapse maturation [17]. Studies have shown that conditional knockout of Dicer in mice, the endoribonuclease that mediates a critical step during miR maturation, drastically affected survival of neural stem cells and decreased differentiation of newborn neurons [18,19,20]. Among the microRNAs (miRs), miR-124 has been reported to play a relevant role in neuronal function, which has been found to be upregulated during neural differentiation [14,21,22]. Forced expression of miR-124 in embryonal carcinoma P19 cells in the presence of retinoic acid promotes a neuronal-like phenotype [20,22,23]. Moreover, an increased expression of miR-124 mediates the conversion of fibroblasts to functional neurons [24,25]. Studies in vivo have further shown that miR-124 was an important regulator of adult neurogenesis in mice [26]. Conversely, knockout of the miR-124-1 gene in mice resulted in defective neuronal survival, impaired hippocampal axonal outgrowth, and reduced brain size [27]. Interestingly, it was been shown that miR-124 targeted the Ezh2 3′-UTR in early neuronal cells, thereby controlling Ezh2 expression and Ezh2-mediated gene silencing during neuron lineage commitment [28]. Additionally, miR-124 targeted and downregulated USP14, coding for a deubiquitinase that binds to Ezh2, and thus prevented ubiquitin-dependent degradation of Ezh2 via proteasome [29,30].

Another relevant microRNA molecule during brain development is let-7 (lethal-7), which has been found in elevated concentrations in this tissue during embryogenesis [31,32,33,34]. Mammals contain a let-7 family, including several members (from let-7a to let-7k), which have been shown to play a significant role in maintaining the balance between neuronal progenitor cell proliferation and commitment to engage neurogenic differentiation, as they control the expression of a number of genes that are critical during this early developmental stages [35,36,37,38]. Interestingly, it has been shown that let-7 mediated control of Ezh2 gene expression in prostate cancer cells [39], as it recognized a specific motif at the Ezh2 3′-UTR. Loss of let-7 function in these tumor cells resulted in increased Ezh2 expression that was accompanied by acquisition of a cancer stem cell signature.

Whereas these data provide support for a regulatory role of miRs over PRC2 expression and function during early neuron lineage commitment, there is a lack of studies that address the contribution of miRs during the maturation of hippocampal neurons, a process that determines the functionality of these cells during learning and memory. Here, we examine whether specific miR molecules contribute to Ezh2 downregulation during hippocampal neuronal maturation. We report that miR-124 and let-7, which are among the group of miRs that are highly expressed in mature hippocampal neurons, can target cognate motifs at the Ezh2 3′-UTR, and thereby dampen Ezh2 expression.

## 2. Results

### 2.1. MicroRNA Expression Profile in Mature Hippocampal Neurons

**Figure 1:**
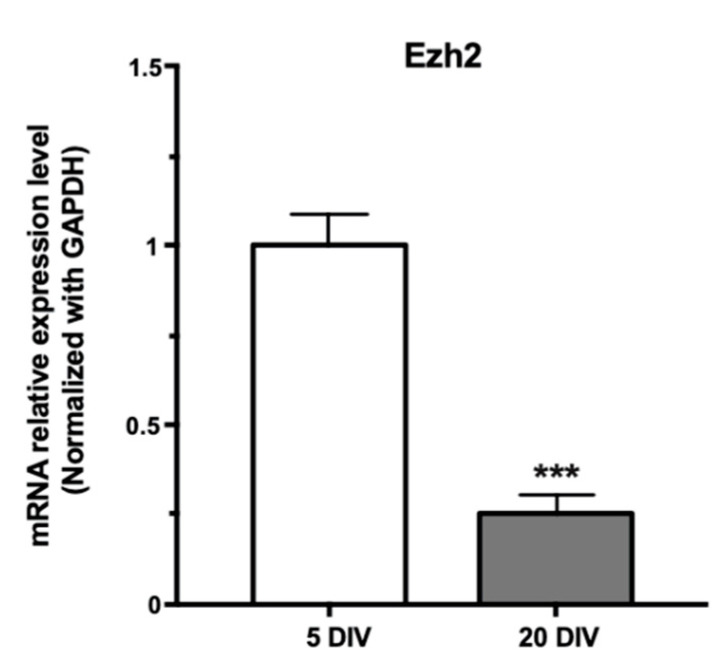
Expression of Enhancer of Zeste Homolog 2 (Ezh2) mRNA is downregulated during hippocampal neuronal maturation. mRNA samples were collected from hippocampal neuron-enriched cultures at 5 and 20 days in vitro (DIV). Then, real-time quantitative PCR (RT-qPCR) analysis was performed to determine the Ezh2 mRNA levels. All values are presented as relative expression normalized to Glyceraldehyde-3-Phosphate Dehydrogenase (GAPDH). (*n* = 3, *** *p* < 0.001).

Previous results have indicated that expression of the methylase Ezh2, the catalytic subunit of the PRC2 complex, was downregulated during hippocampal neuronal maturation [7]. However, the mechanisms that control this reduced expression of Ezh2 (see Figure 1) have not been elucidated.

Accumulating evidence demonstrates that miRs are critical regulators of gene expression in the CNS, controlling key processes that include dendritogenesis and synapse maturation, among others [38]. Hence, we examined the miR expression profile in mature rat hippocampal neurons seeking to identify miR molecules that could mediate this Ezh2 mRNA downregulation (Figure 1). The miRs were isolated from neuron-enriched cell populations obtained from 18-day-old rat embryos (E18) and grown in culture for up to 20 days in vitro (20 DIV), under conditions that significantly reduced glia proliferation and favored neuron maturation [7,40]. The miR populations were harvested from mature (20 DIV) cell cultures, purified, properly labeled, and then analyzed using microarrays (Affimetrix Chip System). The miR expression profile data (see additional data file in Table S1) were analyzed bioinformatically to determine which miR molecules were highly expressed in these mature hippocampal neurons. Importantly, it was detected that, among the top 25 highly expressed miRs (Table 1), there were two families, i.e., miR-124 and let-7, which could potentially recognize the 3′-UTR end of the Ezh2 mRNA in these cells (see below), and therefore potentially contributed to downregulate Ezh2 expression during hippocampal maturation (Figure 1) [7].

### 2.2. Downregulation of Ezh2 Expression during Hippocampal Maturation Requires Dicer

To begin examining the role of miRs on Ezh2 expression in hippocampal neurons, we carried out a knockdown of Dicer expression within these cells, the RNA nuclease that catalyzes the processing of immature miR precursors, thereby allowing the formation of mature and functional miR molecules [41,42]. Two shRNAs coding sequences targeting Dicer mRNA (shDicer1 and shDicer2) were produced as lentiviral vectors, and then infective viral particles were generated. These particles efficiently infected hippocampus neuronal cell types, including the mouse N2A neuroblastoma cell line and primary rat hippocampal neurons, where a significant decrease in Dicer mRNA expression was detected (Figure S1).

**Figure 2:**
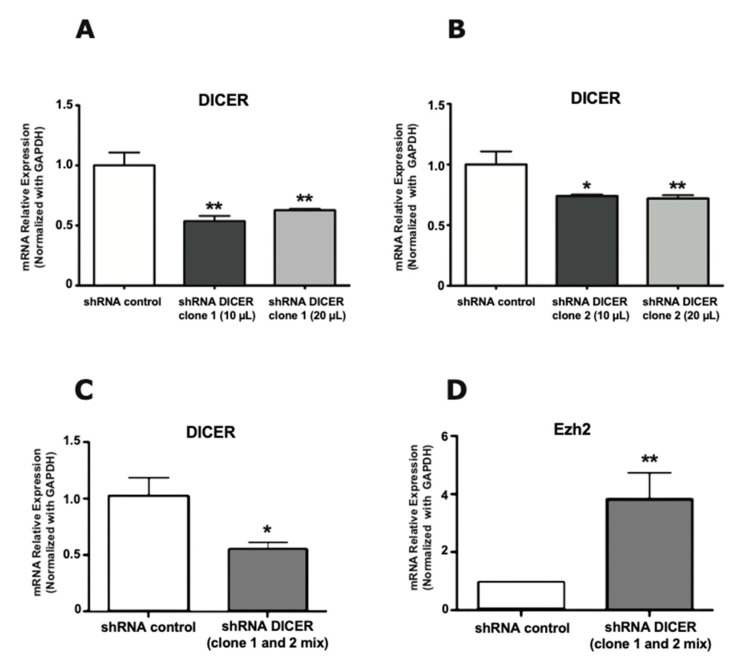
Dicer knockdown in primary mature hippocampal neurons results in enhanced Ezh2 mRNA expression. Reduced Dicer mRNA expression is found after lentiviral transduction of shDicer 1 (**A**) and 2 (**B**) molecules; (**C**) Hippocampal neuron-enriched cultures at 17 DIV were transduced for 72 h with a mix of lentivirus coding shRNA 1 and shRNA 2 against Dicer, and Dicer mRNA expression then analyzed; (**D**) Ezh2 mRNA expression in mature hippocampal neurons (20 DIV) infected with shRNA against Dicer. Results are shown as mean ± SEM (*n* = 3, * *p* < 0.05 and ** *p* < 0.01).

Next, we infected mature (from 17 DIV to 20 DIV) hippocampal neuron-enriched cultures with lentiviruses encoding for the two shRNAs against Dicer. It was found that both molecules downregulated Dicer mRNA levels as compared with an infection with lentiviruses encoding only GFP (Figure 2A, B, respectively). Importantly, we determined that this shRNA-mediated knockdown of Dicer expression (using a mix of both shDicer 1 and 2, Figure 2C) resulted in a significant increase in Ezh2 mRNA levels (Figure 2D). Together, these results indicate that the decreased Ezh2 expression during maturation of hippocampal neurons (Figure 1) can be mediated by miRs that require Dicer for adequate processing and function.

### 2.3. Candidate miRs for Mediating Ezh2 Downregulation Exhibit Increased Expression during Hippocampal Neuronal Maturation

**Figure 3:**
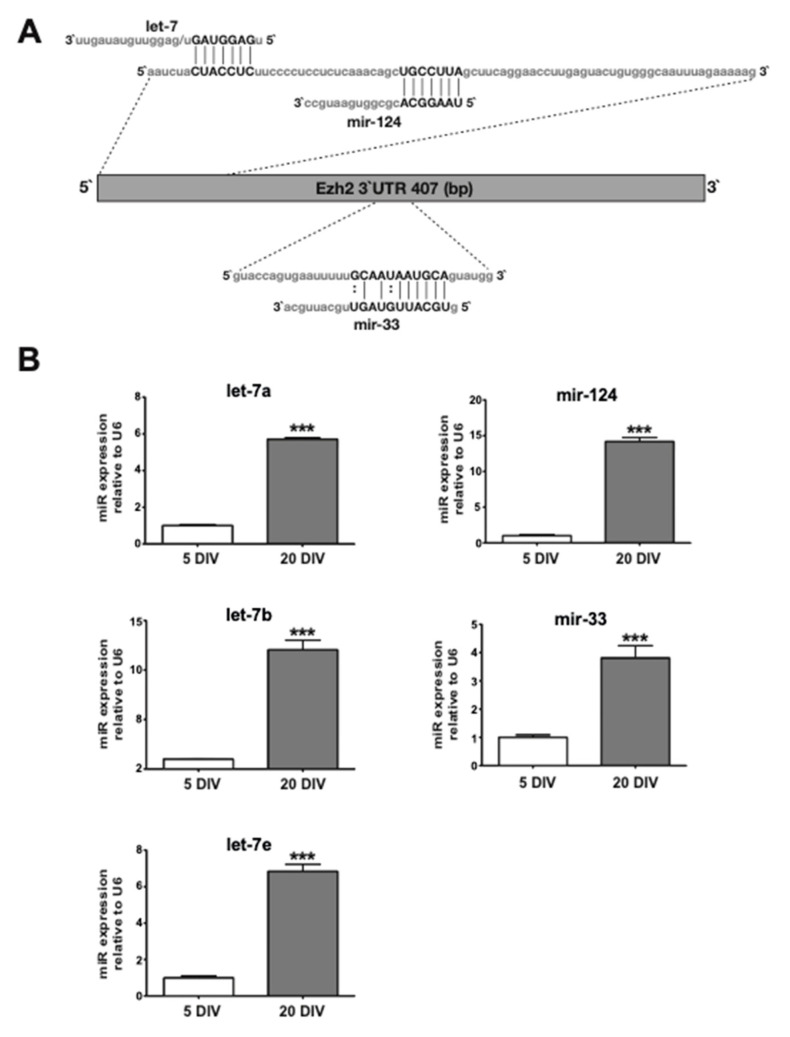
Expression of miRs that potentially bind at the 3′-UTR of Ezh2 mRNAs is upregulated during hippocampal neuronal maturation. (**A**) Schematic representation of Ezh2 3’-UTR and the predicted miR-124, let-7, and miR-33 target sites; (**B**) Quantification of miR-124, let-7 (let-7a, -b, and -e family members) and miR-33 increased expression during hippocampal neuronal maturation (5 to 20 DIV). Each miRNA was quantified using specific TaqMan^®^ probes. Expression of the different miRNAs was normalized against snU6 RNA. Results are shown as mean ± SEM. (*n* = 3, *** *p* < 0.001).

Our highly expressed miR profile in mature hippocampal neurons (Table 1) revealed the presence of miRs that could potentially target the 3′-UTR of the Ezh2 mRNA (see Table 2). Among them, miR-124 has been previously shown to target and downregulate Ezh2 mRNA expression during early mouse neurogenesis [28] and let-7, which has been previously associated with Ezh2 mRNA control in prostate cancer cells [39]. Both miR families could recognize non-overlapping motifs at the Ezh2 3′-UTR (Figure 3A) and were confirmed as upregulated during hippocampal neuronal maturation by using Taqman-based quantitative PCR approaches (see Methods) (Figure 3B). We additionally identified other miRs that could potentially target the Ezh2 3′-UTR. For instance, miR-33 (Figure 3A), although it is detected at relatively lower levels than miR-124 and let-7 members in mature hippocampal neurons (see Table S1), shows an expression that increases during the transition from immature to mature neurons (Figure 3B). It has been shown that miR-33 played a principal role during control of the cholesterol metabolism as it targeted and downregulated the expression of genes associated with the biosynthesis and transport of cholesterol in the liver [43]. Importantly, miR-33 has not been found to be associated with Ezh2 expression control or to play a relevant role in hippocampal neurons.

### 2.4. The 3′-UTR of the Ezh2 Gene Contains Sequence Elements that Mediate Decreased Expression of Immediately Upstream Protein-Coding Genes

**Figure 4:**
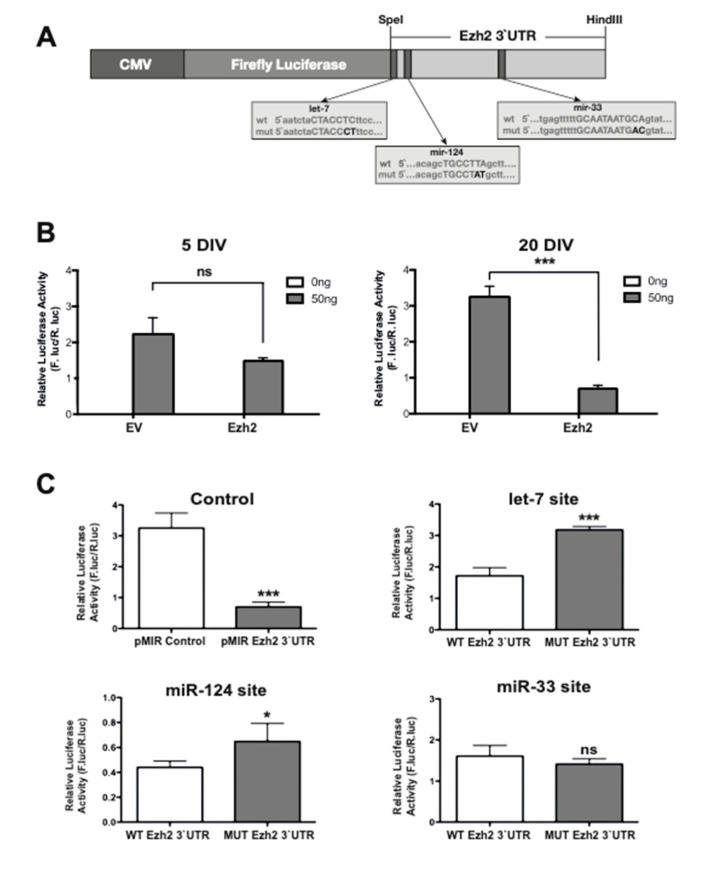
3′-UTR sequence of the Ezh2 gene contains miR motifs that can downregulate the expression of an immediately up-stream luciferase reporter gene in rat hippocampal neurons. (**A**) Schematic representation of the CMV(Cytomegalovirus)-driven luciferase Ezh2 3`-UTR reporter gene (pMIR-report-Ezh2-3′UTR) construct expressed in hippocampal neurons following transient transfection. Wild-type (wt) and mutated (mut) motifs for miR-124, let-7, and miR-33 are shown below the construct. The specific mutations introduced are indicated in black-bold; (**B**) Immature (5 DIV) and mature (20 DIV) hippocampal neurons were transfected with 50 ng of empty vector (EV) or pMIR-report-Ezh2-3′UTR vector and luciferase activity measured 24 h later; (**C**) Relative luciferase activity after expression of wild-type (control) and mutant (indicated at the top of each graph) versions of let-7, miR-124, and miR-33 motifs in mature (20 DIV) hippocampal neurons. Results are shown as mean ± SEM (*n* = 3, * *p* < 0.05 and *** *p* < 0.001).

Next, we examined the ability of the Ezh2 3′-UTR sequence to modulate the expression of an immediately upstream luciferase reporter gene in cells enriched in miRs that can potentially target motifs in this 3′-UTR. For this purpose, the entire Ezh2 3′-UTR segment (407 bp) was cloned downstream of the luciferase reporter gene within the context of Cytomegalovirus (CMV)-driven mammalian expression vector (CMV-LUC-Ezh2-3′UTR, see Figure 4A). This vector was transfected in both immature (5 DIV) and mature (20 DIV) hippocampal neuronal cultures for 24 h, after which the luciferase activity present in the transfected cells was measured. It was found that transfection of this construct in mature neurons resulted in a significantly reduced luciferase activity as compared with that obtained in immature hippocampal cells (Figure 4B). This result indicates that the 3′-UTR of the Ezh2 gene can mediate a decreased biosynthesis of the luciferase protein in mature hippocampal cells that exhibit increased expression of miRs that may potentially target this 3′-UTR sequence.

Next, we examined whether the specific motifs for let-7, miR-124, and miR-33 within the 3′-UTR of the Ezh2 gene were contributing to mediate this downregulation of luciferase expression in mature hippocampal neurons. Specific mutations at each motif were introduced within the context of the entire Ezh2 3′-UTR sequence cloned immediately downstream of the luciferase reporter gene (CMV-LUC-Ezh2-3′UTR vector) (Figure 4A). Each mutated construct was transiently transfected in mature hippocampal neurons (in parallel with the wild-type version of this expression vector) and the effect on the luciferase activity was measured 24 h later. We determined that mutation of the cognate motifs for let-7 and miR-124 prevented the reduced luciferase activity observed with the wild-type version of the construct (Figure 4C). Thus, instead of the decreased luciferase activity (five- to six-fold) produced by the WT Ezh2 3′-UTR construct (control graph), the mutation at the let-7 (let-7 site graph) and miR-124 (miR-124 site graph) elements resulted in higher reporter activity with respect to this wild-type construct (two- and 1.5-fold, respectively). These results suggest that joint or independent binding of endogenous let-7 and miR-124 family members to the Ezh2 3′-UTR sequence can mediate downregulation of the luciferase reporter activity. In addition, mutation of the miR-33 motif resulted in luciferase activity that was comparable to that produced by the wild-type version of the vector (Figure 4C). These results suggest that let-7 and miR-124 motifs, but not the miR-33 motif, can significantly contribute to the Ezh2 3′-UTR-dependent downregulation of Ezh2 expression in mature hippocampal neurons. It is necessary to consider that because mutation of the let-7 and miR-124 motifs did not completely revert the Ezh2 3′-UTR-mediated reduction in the luciferase reporter activity, there could be additional regulatory mechanisms associated with this 3′-UTR sequence that contributed to its inhibitory activity.

### 2.5. Expression of a Let-7 Mimic in Immature Hippocampal Neurons Inhibits Ezh2 Expression

**Figure 5:**
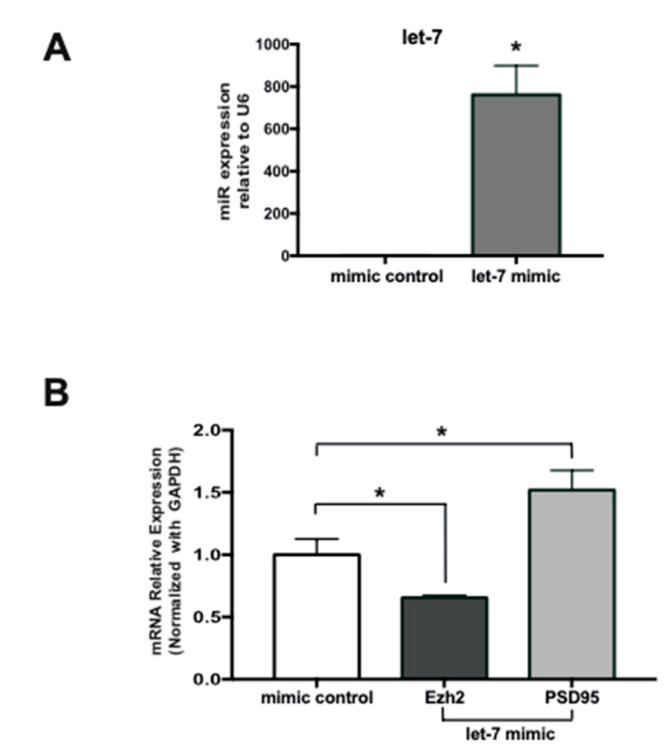
Forced expression of a let-7 mimic downregulates Ezh2 mRNA expression in immature hippocampal neurons. (**A**) Expression of let-7 was significantly higher in immature hippocampal neurons (5 DIV) following a let-7 mimic transfection for 48 h. Quantification was assessed by TaqMan^®^ probes as shown in Figure 3 legend; (**B**) Significantly decreased expression of Ezh2 mRNA follows let-7 mimic transfection in immature hippocampal neurons. In contrast, expression of PSD95/Dlg4 gene mRNA, an Ezh2 target gene in these cells, was found to be significantly enhanced. (*n* = 3, * *p* < 0.05).

To further demonstrate that miRs enriched in mature hippocampal neurons can mediate downregulation of Ezh2 expression, we performed a gain-of-function experiment using a miR mimics-based strategy [44,45]. Because the mutation of the let-7 motif produced the highest effect on the inhibitory activity of the Ezh2 3′-UTR sequence (Figure 4C), we examined the impact of an enhanced expression of let-7e mimics on the levels of the endogenous Ezh2 mRNA in immature (5 DIV) hippocampal neurons. As shown in Figure 5, transfection of these immature neurons with a let-7e mimic results in a robust increase in its expression as compared with a negative control mimic (termed mimic control) (Figure 5A). This let-7 increase is accompanied by a significant reduction in Ezh2 mRNA levels (Figure 5B), further indicating that Ezh2 expression is controlled by miRs that, like let-7, can target the Ezh2 3′-UTR in hippocampal neurons. Importantly, this reduced Ezh2 mRNA expression occurs concomitantly with an increase in PSD95/Dlg4 gene transcription (Figure 5B), a previously reported gene target of the PRC2-Ezh2 complex in immature hippocampal neurons [7].

## 3. Discussion

During early stages of hippocampal development, the repressive activity of the PRC2 complex is critical for both silencing the expression of non-neuronal gene programs (e.g., osteogenic lineage genes) and repressing the expression of neuronal genes coding key synaptic components (e.g., PSD95/Dlg4) that are mainly required at later stages of hippocampal maturation [6,7]. Previous work from our group demonstrated that Ezh2, the catalytic subunit of PRC2, was differentially expressed during maturation of hippocampal neurons; in immature neurons, Ezh2 was abundantly expressed, whereas in mature neurons the expression of Ezh2 was significantly reduced [6,7]. Here, we find that the microRNAs let-7 and miR-124, which are highly enriched in mature hippocampal cells (20 DIV), can mediate downregulation of Ezh2 expression in these cells. Although these data argue in favor of the hypothesis that, during hippocampal maturation, the Ezh2 repressive activity (as part of the PRC2 complex) can be controlled through a miR-mediated post-transcriptional mechanism, it is necessary to consider the contribution of additional mechanisms to this Ezh2 downregulation. These additional mechanisms include changes in histone tail modifications that can inhibit transcription at the Ezh2 gene promoter in hippocampal cells [7] and the proteasome-mediated Ezh2 protein degradation in brain cells [11]. Therefore, it is tempting to propose that Ezh2 expression is tightly controlled during hippocampal maturation by a combination of transcriptional, post-transcriptional, and post-translational mechanisms.

In agreement with the results reported here, miR-124 has been previously found upregulated during mammalian neuronal differentiation [14,21,22] where it contributed to neuronal survival [27]. Moreover, miR-124 has been shown to target the Ezh2 3′-UTR in neuronal cell lines (N2a and P19), thereby downregulating Ezh2 expression and reducing PRC2-Ezh2-mediated gene silencing required for proper progression of neuronal differentiation [28]. Interestingly, miR-124 has also been proposed to be a critical regulator of PRC2-independent functions of Ezh2 during early neural lineage commitment. In this case, miR-124 targeted and downregulated the expression of USP14 [29], a deubiquitinase enzyme that binds to the Ezh2 protein, and prevented its ubiquitin-dependent degradation via proteasome [30]. Together with these previous findings, our results provide further support for the critical role of miR-124 during neuronal function, as miR-124 may control Ezh2 expression at both early and late stages of neuronal differentiation.

The let-7 family members have previously been found to be expressed at elevated concentrations in brain tissues during embryogenesis [31,32,33,34]. However, a direct role of let-7 in hippocampal neuronal differentiation or plasticity has not been reported. Here, we find that a let-7-dependent decrease in Ezh2 mRNA is accompanied by a significant increase in PSD95/Dlg4 mRNA, further supporting the critical role of the PRC2-Ezh2 complex during inhibition of the PSD95/Dlg4 gene transcription in immature hippocampal cells [7]. Because let-7 cannot directly target the PSD95/Dlg4 gene 3′-UTR (let-7 motif is not present at this PSD95/Dlg4 sequence), our results provide a basis for proposing that, in mature hippocampal neurons, let-7 can indirectly promote the expression of relevant hippocampal plasticity components by downregulating the expression of the epigenetic repressor PRC2-Ezh2. These results extend previous findings showing that miR-125, together with fragile X mental retardation protein (FMRP), represses the translation of PSD95 mRNA in hippocampal neurons at early stages (7 DIV) of maturation [46,47].

Future studies must address the specific mechanisms mediating the increased expression of both miR-124 and let-7 during hippocampal neuron maturation. It has been reported that the role of miR-124 during neuronal differentiation was tightly associated with the activity of the Repressor Element-1 Silencing Transcription Factor (REST) [30], a key repressor of neuronal genes in neural progenitors, including sequences coding for the miR-124 RNA precursors [23]. Neuronal differentiation is accompanied with a progressive inhibition of this REST-dependent repression of miR-124 transcription, which results in miR-124 accumulation and subsequent downregulation of target transcripts, including those coding for REST and its associated protein MeCP1, as both transcripts contain miR-124 binding motifs at their 3′-UTRs [23,48]. Whether this type of negative feedback regulatory loop also operates during hippocampal neuronal maturation needs to be established. In the case of let-7, studies should clarify the potential role of the Lin28A/B proteins during control of the let-7 biogenesis in hippocampal neurons [49]. In mouse hippocampal neurons, brain-derived neurotrophic factor (BDNF) has been shown to directly stimulate Lin28 expression in a developing brain, which resulted in an inhibition of let-7 biogenesis (by preventing Dicer-mediated processing of the pre-let-7 transcript and promoting its degradation) and the concomitant increase in let-7 target transcripts, including a minor subset of mRNAs coding for proteins associated with excitatory functions [50]. As BDNF-mediated gene pathways are a relevant component in the physiological function of hippocampal neurons, the contribution of this BDNF-Lin28-miR-124 axis needs to be experimentally addressed.

The contribution of the let-7 and miR-124 motifs in the Ezh2 3′-UTR inhibitory activity appears to be selective and, likely, is related to the elevated concentration of both microRNAs in mature hippocampal cells (both among the top 10 most abundant miRs in these cells). This conclusion is supported by our results indicating that a consensus motif for miR-33, a microRNA expressed at significantly lower relative concentration than let-7 and miR-124 in these mature hippocampal neurons, does not contribute to the Ezh2 3′-UTR-dependent downregulation of the upstream luciferase reporter gene. Previous reports have extensively documented that miR-33 controlled the expression of genes associated with the metabolism of cholesterol, mainly in the liver [43]. Understanding whether this microRNA also regulates specific mRNA targets (different from Ezh2) in hippocampal neurons needs further investigation.

It also remains to be determined whether let-7 and mir-124 functionally collaborate (additively or synergistically) during downregulation of Ezh2 expression in mature hippocampal neurons. Because their specific target motifs are not overlapping at the 3′-UTR sequence of the Ezh2 gene, and because independent mutation of each of these two motifs cannot completely revert the inhibitory activity associated with of this Ezh2 3′UTR sequence, it is reasonable to hypothesize that a collaborative inhibitory mechanism involving both let-7 and miR-124 operates in physiological conditions. Moreover, the possibility that these two molecules function in concert with additional regulatory mechanisms must be addressed in future studies.

## 4. Materials and Methods

### 4.1. Primary Hippocampal Cultures

Protocols of animal management were performed according to NIH guidelines and as approved by the Ethical and Biosafety Committees of Universidad Andres Bello (014/2013; 024/2013; 001/2018). Pregnant Sprague–Dawley rats were deeply anesthetized with CO_2_; hippocampal cultures were prepared from E18 pups, as described previously [7,51,52].

### 4.2. MicroRNA Expression Analyses Using Microarrays

Microarray analyses were performed using 500 ng of miRNA, purified using a mirVana^TM^ miRNA isolation kit (Life Technologies Inc., Ambion, Burlington, ON, Canada). For miRNA labeling, the Flashtaq^TM^ Biotin kit (Genisphere, Hatfield, PA, USA) was used, following the manufacturer′s recommendations. The miRNAs were processed for poly-A tail addition, and then ligated with adapters for biotin labeling and subsequent recognition. Labeled miRNAs were hybridized to GeneChip^TM^ miRNA 2.0 Array (Affymetrix, Santa Clara, CA, USA), which contained 389 probes in triplicate to detect rat miRNAs. Results were analyzed using the software Affymetrix^TM^ miRNA QC Tool.

### 4.3. MicroRNA Quantitation

The miRNAs were extracted using a mirVana^TM^ miRNA isolation kit (Life Technologies Inc.), according to the manufacturer’s instructions. Specific cDNA for each miRNA quantified was synthesized from 10 ng of RNA, using a TaqMan^TM^ MicroRNA Reverse Transcription Kit (Life Technologies Inc.). Then, quantitation was performed for each miRNA using TaqMan^TM^ MicroRNA Assays (Life Technologies Inc.), followed by real-time PCR with TaqMan^TM^ probes.

### 4.4. Plasmid Construction

Reporter plasmids were constructed by inserting the full-length 3′-UTR sequence of Ezh2 (NM_001107051.1) from *Rattus norvegicus* (between Spe I and Hind III sites), downstream of the firefly luciferase gene in the pMIR-report^TM^ vector (Promega, Madison, WI, USA). Ezh2 3′-UTR is 407 bp in length. Mutations were carried out by changing only two nucleotides of each MRE at the 3′-UTR, to prevent broad changes in the 3′-UTR secondary structure. Nucleotide changes were analyzed by using the Mfold (RNA-folding form) web server (http://mfold.rit.albany.edu/?q=mfold/) [53]. The ΔG values for the interactions between each miRNA and MREs were calculated using the RNAhybrid server [54]. Details of the mutations of the MREs generated for each miRNA, are shown in Table 3.

### 4.5. Transient Transfections

Functional 3′-UTR analyses were carried out in cultures of primary rat hippocampal neurons obtained from 18-day-old embryos (E18), that were maintained in Neurobasal^TM^ growth medium (GIBCO, Gaithersburg, MD, USA) supplemented with B27 (GIBCO), 2 mM L-glutamine, 100U/mL penicillin, and 100 μg/mL streptomycin. Transfection was performed at 4 days in vitro (4 DIV) using Lipofectamine^TM^ 3000, following the manufacturer′s instructions (Invitrogen, Carlsbad, CA, USA), and then reporter activity was evaluated at 5 DIV. To evaluate cultures at day 20 DIV, transfection with Neuromag^TM^ was performed at 19 DIV, according to the manufacturer’s instructions (OZ Biosciences, San Diego, CA, USA). Neuronal cultures were transfected with 20 ng of plasmid pMIR-report^TM^ containing the 3′-UTR of the rat Ezh2 gene. Cultures were co-transfected with 10 pg SV-40 pRL Renilla Luciferase control reporter vector, used as a transfection control. These plasmid quantities were selected after performing dose-response curves.

### 4.6. Lentivirus Production and Infection of Hippocampal Neurons

HEK293FT cells were transfected using Lipofectamine^TM^ 3000 reagent (Invitrogen), following the manufacturer’s instructions, with the pVSVg, pΔ8.9, and pLKO.1-shRNA plasmids (at a 1:2:3 ratio, respectively) and a total DNA of 10 μg. TRC1.5 pLKO.1-puro non-mammalian shRNA control plasmid DNA (SHC002, Sigma) was used as negative control. After 12 h, the medium was replaced and cells were maintained at 32 °C for 48 h. Supernatants containing lentiviral particles were collected, filtered through a PVDF filter (0.45 μm pore size), and concentrated by centrifugation at 3800× *g* for 30 min at 4 °C in an Amicon^TM^ Ultra-15 centrifugal filter (100K, Merck Millipore, Burlington, MA, USA), according to the manufacturer’s instructions. Aliquots of concentrated viral particles were stored at −80 °C. Hippocampal cultures evaluated at 20 DIV were infected at 16 DIV with 1.0 × 10^7^ infectious units of virus (IFU)/cell culture plate (30 mm) containing shRNA-Dicer or shRNA-GFP control.

### 4.7. Reverse Transcriptase and Real-Time Quantitative PCR (RT-qPCR)

Total RNA was extracted with TRIzol^TM^ (Invitrogen), according to the manufacturer’s protocol. Then, 2 μg of each sample were used for reverse transcription. The qPCR was performed using SYBR Green^TM^ PCR Master Mix (Applied Biosystems, Life Technologies Inc.). Data are presented as relative mRNA levels of the gene of interest normalized to GAPDH mRNA levels. Primers used were the following: Dicer Fw: GGCAGGTGTACTATCCGATGA, Rev: TGGTTCCATCTCAAGCAATTC; Ezh2 Fw: GCCAGACTGGGAAGAAATCTG, Rev: TCACTGGTGACTGAACACTCC; GAPDH Fw: CATGGCCTTCCGTGTTCCTA, Rev: CCTGCTTCACCACCTTCTTGAT.

### 4.8. Luciferase Reporter Assay

Luciferase activity was measured 24 h after transfection using a Dual-Glo^TM^ Luciferase Assay System (Promega) in a GloMax^TM^ 20/20 Luminometer (Promega, Turner Biosystems, Madison, WI, USA). Firefly luciferase activity was normalized to Renilla luciferase activity to minimize variations in transfection efficiency between experiments. Experiments were performed in triplicate. Quantitative data of the reporter gene assay are presented as mean ± SEM (*n* = 3).

### 4.9. MicroRNA Target Prediction

To identify miRNAs that potentially target the 3′-UTR of Ezh2 mRNAs, we used the following miRNA target prediction sites: TargetScan (http://www.targetscan.org/) [55] and miRDB (http://mirdb.org/) [56].

### 4.10. Expression of Let-7 Mimics

For the in vitro transfection of immature hippocampal neurons, the transfection mix was prepared using 90 pmol of let-7 mimic (mirVana^TM^ miRNA mimic, Life Technologies) and 15 μL of Lipofectamine^TM^ RNAiMax (Invitrogen), according to the manufacturer′s protocol, for 48 h. The transfection was confirmed by RT-qPCR. Each experiment was repeated at least 3 times.

### 4.11. Statistical Analysis

For the expression analysis we used Student’s t-test to determine significant differences between experiments. In all figures, error bars represent the mean ± standard error of the mean; * *p* < 0.05, ** *p* < 0.01, and *** *p* < 0.001.

## Figures and Tables

**Table 1:** List of top 25 highly expressed microRNAs in mature hippocampal neurons. The data was originated by microarray analyses from three independent experiments (*n* = 1, 2, and 3).

ProbeSetID	20 DIV n1	20 DIV n2	20 DIV n3	Mean 20 DIV
rno-miR-125b-5p_st	14,19586735	14,11524634	14,02201945	14,1110444
rno-let-7c_st	14,05654371	13,7811261	14,33908404	14,058918
rno-miR-124_st	13,14528831	14,67637917	13,74687013	13,8561792
rno-let-7b_st	13,77215925	13,48533913	14,02162067	13,7597064
hp_rno-mir-124-3_s_st	12,66468198	14,17408833	13,4111353	13,4166352
hp_rno-mir-124-1_s_st	12,55263545	14,12533502	13,40486244	13,3609443
hp_rno-mir-124-2_s_st	12,50969099	13,9984208	13,40293019	13,3036807
rno-miR-103_st	12,86020989	13,58644686	13,02428652	13,1569811
rno-miR-99a_st	13,4265623	13,03790836	12,6824616	13,0489774
rno-miR-107_st	12,54827077	13,33459961	12,92074488	12,9345384
rno-miR-16_st	13,06704109	13,08511435	12,45829264	12,8701494
rno-miR-138_st	11,97407534	13,46384005	13,02131292	12,8197428
rno-miR-24_st	13,22507587	12,5403456	12,45652393	12,7406485
rno-miR-22_st	12,80712275	12,92439752	12,4873622	12,7396275
rno-let-7a_st	12,7448344	12,65429313	12,66395779	12,6876951
rno-miR-181a_st	12,18081162	13,05708698	12,71542614	12,6511082
rno-miR-191_st	12,97155739	12,54972548	12,42775979	12,6496809
rno-miR-23a_st	13,4611495	12,41864159	12,06288726	12,6475594
rno-let-7d_st	12,33069725	12,736612	12,86714228	12,6448172
rno-miR-26a_st	12,50207374	12,94821494	12,21461208	12,5549669
rno-let-7e_st	12,13864311	12,47117626	12,48884926	12,3662229
rno-miR-125a-5p_st	12,67967	12,30327676	11,86511822	12,2826883
rno-miR-130a_st	12,38279452	12,62256525	11,75596603	12,2537753
rno-miR-127_st	11,38137317	12,79650642	12,28065679	12,1528455
rno-miR-99b_st	12,31702088	11,70084872	11,9723612	11,9967436

**Table 2:** The microRNA (miR) families that can potentially target the 3′-UTR region of Ezh2 mRNAs in mature rat hippocampal neuron-enriched cultures (20 DIV). Information about the specific chromosome location of each miR family member is provided. The position of miRs within a sequence that clusters additional microRNA- and other-coding genes is also indicated. IR, intergenic region.

Target	microRNA	Chromosome	Coding Details
	let-7a-1	17	Clustered miR-3596b, miR-3596d, let-7d; let-7f-1
	let-7a-2	8	Clustered miR-100, miR-3695a, in lnc215 gene
	let-7b	7	Clustered let-7c-2
	let-7c-1	11	Clustered miR-99a
	let-7c-2	7	Clustered let-7b
	let-7d	17	Clustered miR-3596b, miR-3596d, let-7a-1, let-7f-1, in Spaca6 gene
	let-7e	1	Clustered miR-99b, miR-3596c, miR-125a
Ezh2	let-7f-1	17	Clustered 3596b, miR-3596d, let-7a-1, let-7d
	let-7f-2	X	Clustered miR-98, in Huwe1 gene
	let-7g	8	IR
	let-7i	7	IR
	miR-124-1	15	IR
	miR-124-2	2	IR
	miR-124-3	3	IR
	miR-33	7	In Srebf2 gene

**Table 3:** Mutations performed at miR-124, let-7, and miR-33 motifs within the Ezh2 3′-UTR region. Specific mutated nucleotides for each miRNA recognition element (MRE) are indicated in bold black. Changes in the predicted **Δ**G values for both wild-type (wt) and mutated miR motifs are also included.

miR Analyzed	Ezh2 3′UTR	MRE—miR Alignments Predicted	ΔG MRE WT	ΔG MRE MUT
**WT**		**5**′AAUCUACUACCUCU **3**′		
**let-7**	7–13	**3**′UGAUAUGUUGGAGGAUGGAGU **5**′	−20.1	−15.4
**MUT**		**5**′AAUCUACUACC**CU**U **3**′		
**WT**		**5**′CUCCUCUCAAACAGCUGCCUUAG **3**′		
**miR-124**	33–39	**3**′CCGUAAGUGGCGC-ACGGAAU **5**′	−19.9	−15.8
**MUT**		**5**′CUCCUCUCAAACAGCUGCCU**AU**G **3**′		
**WT**		**5**′UGAAUUUUUGCAAUAAUGCAG **3**′		
**miR-33**	195–216	**3**′ACGUUACGUUGAUGUUACGUG **5**′	−18.2	−14.2
**MUT**		**5**′UGAAUUUUUGCAAUAAUG**AC**G **3**′		

## Supplementary Materials

The following are available online at https://www.mdpi.com/1422-0067/21/22/8472/s1.

## Abbreviations

miRs	MicroRNAs
CNS	Central nervous system
IFU	Infectious units of virus
FMRP	Fragile X mental retardation protein
BDNF	Brain-derived neurotrophic factor
