# Supplementary material for: Downregulation of the Polycomb-Associated Methyltransferase Ezh2 during Maturation of Hippocampal Neurons Is Mediated by MicroRNAs Let-7 and miR-124
Source: Int J Mol Sci. 2020 Nov 11;21(22):8472. doi: 10.3390/ijms21228472 (PMC7697002; doi:10.3390/ijms21228472)
Supplement: Supplementary file 1 [file ijms-21-08472-s001.pdf]

Supplementary Figure S1.-

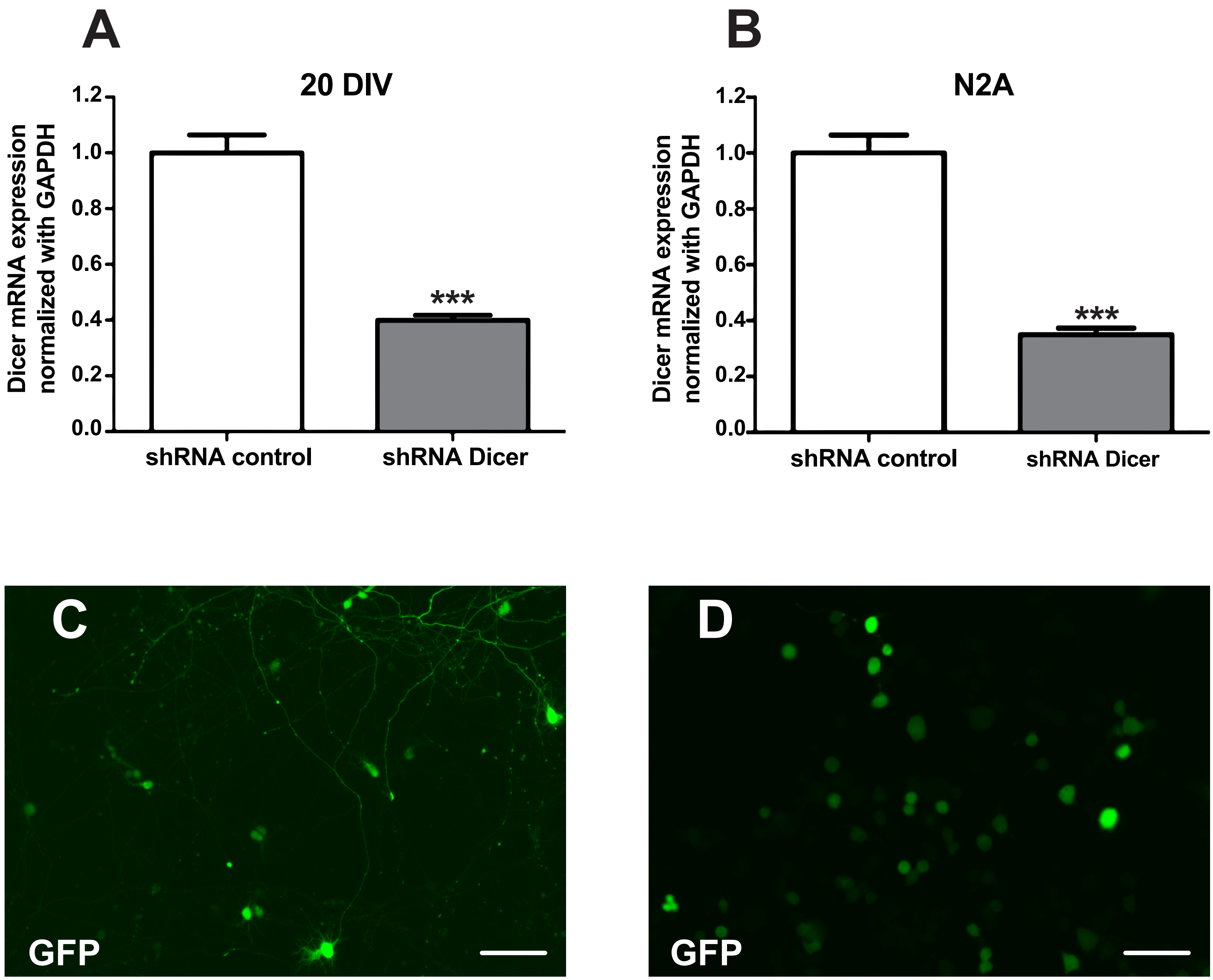

Figure S1.- Dicer knockdown in primary hippocampal neurons and the hippocampal N2A cell line. Decreased Dicer mRNA expression after infection for 72 h with a lentivirus coding a shRNA against Dicer in mature (17 to 20 DIV) rat hippocampal neuron cultures (A) and in the N2A rat hippocampal cell line (B). To confirm efficient infection rates, primary rat hippocampal neurons (C) and N2A cells (D) were also transduced with a lentivirus coding for a shRNA against the Dicer mRNA and for the GFP protein. GFP positive cells data indicate that more than 80% of the neurons and N2A cells were infected. n=3, ***p<0.001. Bars represent 50 µm.

Supplementary data file S1.- miRNA expression analyses using microarrays (Affymetrix) in samples from mature (20 DIV) hippocampa

ProbeSetID	20DIV1_(miRNA-2_0)	20DIV2_(miRNA-2_0)	20DIV3_(miRNA-2_0)	Mean_20DIV
rno-miR-125b-5p_st	14,19586735	14,11524634	14,02201945	14,11104438
rno-let-7c_st	14,05654371	13,7811261	14,33908404	14,05891795
rno-miR-124_st	13,14528831	14,67637917	13,74687013	13,8561792
rno-let-7b_st	13,77215925	13,48533913	14,02162067	13,75970635
hp_rno-mir-124-3_s_st	12,66468198	14,17408833	13,4111353	13,41663521
hp_rno-mir-124-1_s_st	12,55263545	14,12533502	13,40486244	13,3609443
hp_rno-mir-124-2_s_st	12,50969099	13,9984208	13,40293019	13,30368066
rno-miR-103_st	12,86020989	13,58644686	13,02428652	13,15698109
rno-miR-99a_st	13,4265623	13,03790836	12,6824616	13,04897742
rno-miR-107_st	12,54827077	13,33459961	12,92074488	12,93453842
rno-miR-16_st	13,06704109	13,08511435	12,45829264	12,87014936
rno-miR-138_st	11,97407534	13,46384005	13,02131292	12,81974277
rno-miR-24_st	13,22507587	12,5403456	12,45652393	12,74064846
rno-miR-22_st	12,80712275	12,92439752	12,4873622	12,73962749
rno-let-7a_st	12,7448344	12,65429313	12,66395779	12,68769511
rno-miR-181a_st	12,18081162	13,05708698	12,71542614	12,65110825
rno-miR-191_st	12,97155739	12,54972548	12,42775979	12,64968089
rno-miR-23a_st	13,4611495	12,41864159	12,06288726	12,64755945
rno-let-7d_st	12,33069725	12,736612	12,86714228	12,64481717
rno-miR-26a_st	12,50207374	12,94821494	12,21461208	12,55496692
rno-let-7e_st	12,13864311	12,47117626	12,48884926	12,36622288
rno-miR-125a-5p_st	12,67967	12,30327676	11,86511822	12,28268833
rno-miR-130a_st	12,38279452	12,62256525	11,75596603	12,25377526
rno-miR-127_st	11,38137317	12,79650642	12,28065679	12,15284546
rno-miR-99b_st	12,31702088	11,70084872	11,9723612	11,9967436
rno-miR-23b_st	11,9145411	12,13498944	11,83949935	11,96300996
rno-miR-30c_st	11,8384657	11,86677518	11,59147672	11,76557253
rno-miR-132_st	10,77089728	12,22169572	11,96920301	11,653932
rno-miR-221_st	11,28158653	12,21972124	11,43994663	11,6470848
rno-miR-100_st	11,71318303	11,46168645	10,91730487	11,36405811

rno-let-7i_st	10,79075063	11,67753337	11,25156246	11,23994882
rno-miR-222_st	10,77335231	11,76764832	10,94226552	11,16108871
rno-miR-34a_st	11,64628346	11,22173435	10,60932769	11,15911517
rno-miR-151_st	11,134957	11,25359759	11,08798088	11,15884515
rno-miR-128_st	10,42944794	11,80921251	10,84831018	11,02899021
rno-miR-487b_st	10,0918823	11,44022714	11,44177589	10,99129511
rno-miR-181b_st	10,13984232	11,37346744	11,19269991	10,90200322
rno-miR-185_st	10,29298536	11,65874693	10,6309665	10,8608996
rno-miR-320_st	10,58876048	10,82275423	10,93720656	10,78290709
rno-miR-30a_st	10,52966843	11,05843852	10,1917611	10,59328935
rno-miR-93_st	10,65259278	10,29447258	10,77160293	10,57288943
rno-miR-29a_st	10,97542588	11,44315006	9,099482728	10,50601956
rno-miR-361_st	10,07495719	10,85047181	10,31333641	10,4129218
rno-miR-27a_st	10,86514395	11,12033936	9,245830753	10,41043802
rno-miR-382_st	9,653069487	10,91556202	10,63748409	10,40203853
rno-miR-342-3p_st	10,00953847	10,70931428	10,40294493	10,37393256
rno-miR-434_st	9,780529456	10,63903359	10,29172801	10,23709702
rno-miR-449a_st	10,8151309	9,746083823	10,04301869	10,20141114
v11_rno-miR-17_st	10,1202755	10,34382341	10,09868503	10,18759465
rno-miR-17-5p_st	10,12824305	10,35072704	10,02927855	10,16941622
rno-miR-674-5p_st	9,980690685	10,35971382	10,13517065	10,15852505
rno-miR-9-star_st	9,636051284	10,87719632	9,903387075	10,13887822
rno-miR-344-3p_st	9,41819075	10,92438648	9,981021088	10,1078661
rno-miR-378_st	10,54913685	10,01384878	9,71403889	10,09234151
rno-miR-324-5p_st	9,890045611	10,86508397	9,481282216	10,07880393
rno-miR-34c_st	10,07480205	10,41248378	9,456136991	9,98114094
rno-miR-145_st	10,38281979	9,088321767	10,44127592	9,970805827
rno-miR-30b-5p_st	9,910883105	10,61230353	9,3344307	9,952539111
rno-miR-187_st	9,69453121	9,975843416	10,11487154	9,928415388
rno-miR-541_st	8,511581051	10,66789383	10,1793881	9,786287661
rno-miR-140-star_st	9,636824861	10,25536912	9,441547899	9,77791396
rno-miR-433_st	8,515170364	10,2964829	10,37817058	9,729941282
rno-miR-379_st	8,118704547	11,02651909	9,978173341	9,707798994

rno-miR-425_st	9,724028415	9,95770504	9,196402341	9,626045265
rno-miR-30d_st	9,7015287	9,987387766	9,122069066	9,603661844
rno-miR-129-star_st	8,498584735	9,780859994	10,22545122	9,501631983
rno-miR-181d_st	8,866261556	10,12226048	9,456764241	9,481762092
rno-miR-195_st	9,291921552	10,42805518	8,698156864	9,472711199
rno-miR-134_st	8,611306503	9,61709142	10,06394114	9,430779689
rno-miR-298_st	8,85984877	9,908807764	9,357704456	9,375453663
rno-miR-708_st	8,850143977	10,50811866	8,689393684	9,349218774
rno-miR-20a_st	9,511851112	9,557683332	8,894040783	9,321191743
rno-miR-151-star_st	9,297284014	9,212805142	9,44142303	9,317170728
rno-miR-325-5p_st	8,658260946	9,833856176	9,259839768	9,250652297
rno-miR-672_st	8,253467002	9,478993465	9,845542059	9,192667508
rno-miR-129_st	8,114749794	9,697329942	9,748898289	9,186992675
rno-miR-212_st	8,676816445	9,620147885	9,064535385	9,120499905
rno-miR-770_st	8,029355784	9,365215097	9,944114654	9,112895178
rno-let-7f_st	8,235803588	10,1256176	8,954898354	9,105439846
rno-miR-346_st	8,525335052	8,556736608	10,21224659	9,098106084
rno-miR-27b_st	8,836320041	9,850648359	8,555716222	9,080894874
rno-miR-125b-star_st	8,730326979	9,493944344	8,851660552	9,025310625
rno-miR-31_st	9,074875355	9,616711208	8,348148648	9,013245071
rno-miR-92b_st	9,38656336	8,420265232	9,188573303	8,998467298
rno-miR-106b_st	8,840353809	9,607010383	8,530381809	8,992582
rno-miR-143_st	8,902248263	8,669902497	9,07706616	8,883072307
rno-miR-652_st	9,375812825	8,893100285	8,268099494	8,845670868
rno-miR-497_st	7,77109655	9,717094396	8,602765578	8,696985508
rno-miR-1224_st	10,37615893	6,618809483	9,069416404	8,688128272
rno-miR-300-3p_st	7,288800981	9,771488702	8,950937983	8,670409222
rno-miR-411_st	7,241526056	10,2352987	8,230818601	8,569214451
rno-miR-500_st	8,113663771	8,915399399	8,45514641	8,494736527
rno-miR-383_st	7,666816374	9,021028809	8,69239798	8,460081054
rno-miR-324-3p_st	8,231367641	8,554523155	8,488145119	8,424678638
rno-miR-138-star_st	7,698806724	9,283174597	8,170865168	8,384282163
rno-miR-135a-star_st	8,081352466	8,312779728	8,538270863	8,310801019

rno-miR-329_st	7,366202505	9,149720274	8,394216869	8,303379882
rno-miR-139-5p_st	7,576751738	9,273614859	7,901342841	8,250569813
rno-miR-345-5p_st	8,461453719	8,202836896	8,061812919	8,242034511
rno-miR-330-star_st	7,598731284	8,828116523	8,291505787	8,239451198
rno-miR-125b-3p_st	8,425000113	8,122670715	8,169939022	8,239203284
rno-miR-19b_st	7,82086243	9,090264722	7,761499884	8,224209012
rno-miR-532-5p_st	7,65392489	8,932704809	8,064456737	8,217028812
rno-miR-331_st	7,462327071	8,84974023	8,175974064	8,162680455
rno-miR-764_st	9,252627771	4,483007892	10,66497194	8,133535868
rno-miR-351_st	10,03505044	8,163618048	6,143213637	8,113960709
rno-miR-152_st	9,126258038	9,181829188	5,954383178	8,087490135
rno-miR-466b_st	8,409964303	5,241613076	10,55224085	8,06793941
rno-miR-204_st	8,774029184	8,38560544	6,925430299	8,028354974
rno-miR-146b_st	7,399248241	8,827587384	7,856905083	8,02791357
rno-miR-92a_st	8,857556754	7,532560664	7,672553908	8,020890442
rno-miR-760-3p_st	7,780447765	7,787649479	8,435677677	8,001258307
rno-miR-341_st	7,170901474	8,819094556	7,827567422	7,939187817
rno-miR-34c-star_st	8,507841793	7,763763343	7,436342733	7,90264929
rno-miR-328_st	7,771998363	7,245439399	8,644076788	7,887171517
rno-miR-194_st	7,514173874	9,07328017	7,029162559	7,872205535
rno-miR-301a_st	7,366267248	8,859515261	7,332082266	7,852621592
rno-miR-296_st	7,98390783	8,028435574	7,422987143	7,811776849
rno-miR-181c_st	7,188812393	9,455242023	6,704969417	7,783007944
rno-miR-204-star_st	7,923785313	7,80624057	7,459817004	7,729947629
rno-miR-494_st	6,961490659	8,331381972	7,795324669	7,696065767
rno-miR-199a-5p_st	9,029729991	8,079856852	5,964946914	7,691511252
rno-miR-30e_st	7,239773243	8,960883955	6,819848727	7,673501975
rno-miR-25_st	8,223659072	7,97019934	6,825806318	7,673221577
rno-miR-551b_st	6,099110865	9,304425434	7,585496097	7,663010799
rno-miR-99b-star_st	7,475653877	7,661795565	7,675342466	7,604263969
rno-miR-532-3p_st	7,895729026	7,253809255	7,58295343	7,577497237
rno-miR-24-2-star_st	8,0653455	8,170949986	6,449031156	7,561775547
rno-miR-485_st	6,325492369	8,332645354	7,937367926	7,531835216

rno-miR-376a_st	6,58088311	9,614859135	6,389275549	7,528339265
rno-miR-15b_st	7,504756648	8,325273355	6,750894792	7,526974932
rno-miR-154_st	6,447188295	8,725606526	7,300312557	7,491035793
rno-miR-410_st	6,456836178	8,530165298	7,441445121	7,476148866
rno-miR-297_st	8,175792631	4,459567873	9,722383855	7,452581453
rno-miR-504_st	6,220712439	8,107256575	7,95046882	7,426145944
rno-miR-30a-star_st	7,663023481	7,688580386	6,731598606	7,361067491
rno-miR-210_st	7,453114894	10,61622199	3,971297036	7,346877972
rno-miR-668_st	6,268062242	7,58148764	7,992469427	7,280673103
rno-miR-214_st	8,577949258	7,527982999	5,685960645	7,263964301
hp_rno-mir-346_st	7,318867341	5,909759174	8,552878011	7,260501509
rno-miR-423_st	7,786079657	7,402247899	6,56108554	7,249804365
rno-miR-9_st	6,456984784	8,255093652	6,831870841	7,181316426
rno-miR-181a-star_st	6,752820694	8,214576052	6,276421132	7,081272626
rno-miR-342-5p_st	6,179651865	8,047876011	6,983698087	7,070408654
rno-miR-199a-3p_st	8,673581322	8,253143486	4,197040837	7,041255215
rno-miR-370_st	5,687374251	7,961728925	7,377712636	7,008938604
rno-miR-409-3p_st	6,39805101	7,417122371	7,156032864	6,990402081
rno-miR-323_st	6,794784088	7,660962207	6,405237951	6,953661415
rno-miR-874_st	7,460983601	7,01312534	6,204936513	6,893015151
rno-miR-28_st	7,626205299	7,581486346	5,406808822	6,871500155
rno-miR-25-star_st	7,242326634	6,272481172	7,075793229	6,863533678
rno-miR-29b-2-star_st	6,144757873	7,940358431	6,391980028	6,825698777
rno-miR-362_st	6,416666213	7,457507922	6,488935219	6,787703118
rno-miR-935_st	5,419164327	7,58968041	7,273415968	6,760753569
rno-miR-339-5p_st	7,271155213	6,895824268	6,111175781	6,759385087
rno-miR-139-3p_st	6,646168135	6,732387641	6,759944459	6,712833412
rno-miR-125a-3p_st	6,511337805	7,008919643	6,417644496	6,645967315
hp_rno-mir-935_st	6,499850741	5,961637253	7,273349246	6,57827908
rno-miR-674-3p_st	7,120069632	7,149757912	5,464716733	6,578181426
rno-miR-667_st	6,395069325	5,866451182	7,373710388	6,545076965
rno-miR-352_st	5,961788938	7,349432018	6,31417035	6,541797102
rno-miR-540_st	5,399739937	7,717633338	6,419781083	6,512384786

rno-miR-350_st	5,989502377	7,406193397	6,084477962	6,493391245
rno-miR-30c-2-star_st	6,186085846	6,938689201	6,25717551	6,460650186
rno-miR-665_st	5,865965068	7,326372979	6,173706635	6,455348227
rno-miR-124-star_st	4,91107626	8,148387175	6,233126607	6,430863347
rno-miR-376b-3p_st	5,643376498	8,211669725	5,348685385	6,40124387
rno-miR-345-3p_st	6,168272389	6,848751606	6,160057489	6,392360495
rno-miR-192_st	6,358101998	7,825663446	4,926798993	6,370188146
rno-miR-431_st	4,874994786	7,592339057	6,573444502	6,346926115
rno-miR-673_st	5,279671425	6,239029165	7,247867846	6,255522812
rno-miR-337_st	4,607415056	7,522229098	6,601426137	6,243690097
rno-miR-18a_st	6,173135186	6,951048089	5,549286314	6,224489863
rno-miR-598-3p_st	5,38293032	5,649586707	7,490517133	6,17434472
rno-miR-493_st	6,003922375	5,292672762	7,193219913	6,163271683
rno-miR-495_st	4,452106298	7,539318179	6,229716106	6,073713527
rno-miR-106b-star_st	6,708138993	6,740562303	4,742854832	6,063852042
rno-miR-330_st	4,961395157	7,734940617	5,486935203	6,061090326
rno-miR-326_st	5,928321398	7,069606013	5,184401811	6,060776407
rno-miR-369-5p_st	5,380706594	8,160259939	4,531668436	6,024211657
rno-miR-539_st	4,688982083	7,48492339	5,850703634	6,008203036
rno-miR-29b_st	6,738750075	8,78297762	2,34566057	5,955796089
rno-let-7d-star_st	5,388003361	5,17513709	7,229651398	5,930930617
rno-miR-666_st	6,198927792	5,732849083	5,796194234	5,909323703
hp_rno-mir-423_st	6,111934451	5,903022266	5,648314628	5,887757115
rno-miR-466c_st	6,311036024	3,530428144	7,614618142	5,818694103
rno-miR-140_st	6,54224475	7,60860493	3,191013553	5,780621078
rno-miR-339-3p_st	5,545603935	6,349611194	5,375090026	5,756768385
rno-miR-877_st	6,035400595	5,423333562	5,791063785	5,749932647
hp_rno-mir-17-1_st	4,585338593	5,122438728	7,502727926	5,736835083
rno-miR-291a-3p_st	6,119443007	0,969903018	9,391079413	5,493475146
rno-miR-146a_st	6,954123169	7,876638676	1,621750956	5,484170934
rno-miR-378-star_st	7,015838114	6,254384299	3,052233342	5,440818585
rno-miR-207_st	5,789315221	3,091032493	7,42242609	5,434257935
rno-miR-758_st	3,827019065	6,359839897	6,008186861	5,398348608

rno-miR-206_st	4,056039001	6,838206921	5,295615131	5,396620351
hp_rno-mir-351_st	5,731430612	5,145463988	5,202708721	5,359867774
rno-miR-384-5p_st	4,658135187	7,700396115	3,502246633	5,286925978
rno-miR-338-star_st	5,065915605	6,234621451	4,525076603	5,275204553
hp_rno-mir-207_st	5,930591229	3,676631071	6,155729788	5,254317363
rno-miR-543-star_st	4,302654238	6,603462995	4,743443463	5,216520232
hp_rno-mir-17-1_x_st	5,261986865	4,870994689	5,428650184	5,187210579
rno-miR-503_st	7,0960943	6,635246639	1,81120444	5,18084846
rno-miR-678_st	6,867195613	3,515142663	5,085761252	5,156033176
rno-miR-28-star_st	6,866835324	6,453853038	2,090048041	5,136912134
hp_rno-mir-339_st	5,380706594	3,716284223	6,224681675	5,107224164
rno-miR-465_st	6,282285411	2,897526479	6,120148127	5,099986672
rno-miR-130b_st	5,309272638	6,212353556	3,698653723	5,073426639
rno-miR-193-star_st	5,695331119	5,854995391	3,649557712	5,066628074
rno-miR-17-3p_st	5,004905903	6,461010165	3,689696868	5,051870979
hp_rno-mir-185_st	4,607359444	5,510569956	4,791153743	4,969694381
rno-miR-873_st	3,322533142	7,201134688	4,158635694	4,894101175
rno-miR-34b_st	5,492076069	6,672336424	2,442754025	4,869055506
rno-miR-126_st	4,535400497	6,877466028	3,051275443	4,821380656
rno-miR-323-star_st	4,205016078	6,024166989	4,12934383	4,786175632
rno-miR-322-star_st	6,453853038	5,656141374	2,168148881	4,759381098
rno-miR-22-star_st	5,48318955	6,584868026	2,168839687	4,745632421
rno-miR-23a-star_st	5,549541016	3,960458235	4,710494016	4,740164422
rno-miR-30e-star_st	4,656258129	6,33684635	3,18527632	4,726126933
rno-miR-872-star_st	5,824546408	6,882566189	1,460334323	4,722482307
hp_rno-mir-139_st	4,950299696	4,562877085	4,582586071	4,698587617
rno-miR-188_st	3,753909331	5,903137662	4,334708759	4,663918584
hp_rno-mir-9-3_st	4,045077894	4,218236849	5,707631411	4,656982051
rno-miR-29c_st	4,788713617	7,315889622	1,859193022	4,654598754
rno-miR-409-5p_st	2,989314371	6,389716631	4,480839021	4,619956674
rno-miR-542-5p_st	6,590947748	6,128768263	1,061758776	4,593824929
rno-miR-375_st	4,969727248	5,753940711	2,713374196	4,479014051
rno-miR-137_st	3,43314801	7,921121252	2,058396332	4,470888531

rno-miR-21_st	5,966246613	6,306123972	0,90145373	4,391274772
rno-miR-412_st	2,5886143	5,575796309	4,829426218	4,331278942
rno-miR-343_st	4,774063367	2,917934227	4,921075406	4,204357667
rno-miR-335_st	3,680836802	6,337355909	2,573843017	4,197345243
rno-miR-29c-star_st	4,236506383	6,164395316	2,09286719	4,16458963
rno-miR-598-5p_st	3,930540535	5,082531874	3,473490222	4,162187544
rno-miR-455_st	4,824593209	5,912990823	1,468905262	4,068829765
rno-miR-148b-3p_st	4,154255966	6,302143218	1,614788999	4,023729395
rno-miR-30c-1-star_st	3,680836802	5,333781893	3,051275443	4,021964713
rno-miR-218-star_st	3,26145796	5,987007746	2,687790458	3,978752055
rno-miR-380_st	2,354028697	6,777565225	2,693681572	3,941758498
rno-miR-299_st	3,847297013	5,870789857	1,989812967	3,902633279
rno-let-7b-star_st	4,684232316	2,597180733	4,349543872	3,87698564
rno-miR-205_st	3,234372104	5,290336432	3,07256889	3,865759142
rno-miR-615_st	6,000668656	2,448803306	2,844264923	3,764578962
hp_rno-mir-667_st	3,967560007	3,225462516	3,927252398	3,706758307
rno-miR-7a_st	1,174091217	6,748586903	3,166124803	3,696267641
hp_rno-mir-425_st	3,985447105	4,005873256	2,949505998	3,64694212
rno-miR-218_st	2,051867346	6,244640776	2,615915942	3,637474688
rno-miR-496_st	2,397136411	5,386461653	2,966258279	3,583285448
rno-miR-98_st	2,484281624	5,663322233	2,546079431	3,564561096
rno-miR-7a-star_st	2,896033503	5,829100035	1,81120444	3,512112659
rno-miR-193_st	3,544933788	5,116479802	1,855401107	3,505604899
rno-miR-708-star_st	2,110331978	5,626940829	2,724799618	3,487357475
rno-miR-153_st	2,813844837	6,473051885	1,150495118	3,479130613
hp_rno-mir-24-2_x_st	3,897606997	2,9665364	3,559192025	3,474445141
rno-miR-150_st	4,925646958	3,267574245	2,185124964	3,459448723
hp_rno-mir-99b_st	3,707847897	3,457600279	3,207600497	3,457682891
rno-miR-377_st	2,78917312	6,118148359	1,40067136	3,435997613
rno-miR-101b_st	2,442149719	5,276691757	2,576949971	3,431930482
hp_rno-mir-770_st	3,100531675	3,351993297	3,806121272	3,419548748
rno-miR-338_st	2,759732164	6,218327166	1,147038823	3,375032717
hp_rno-mir-324_st	3,812716002	3,172362328	3,098467105	3,361181812

rno-miR-322_st	3,917689012	4,787163797	1,371249687	3,358700832
rno-let-7e-star_st	3,442674078	4,537924752	2,077111766	3,352570199
hp_rno-mir-9-3_x_st	2,942636557	3,293565978	3,770800046	3,335667527
hp_rno-mir-674_x_st	3,374750816	3,456234324	3,06314568	3,298043607
rno-miR-20b-5p_st	2,576875367	5,501370338	1,813546371	3,297264025
rno-miR-671_st	2,345525529	4,512375339	2,979659	3,279186622
hp_rno-mir-337_st	2,233432194	3,99601177	3,57007743	3,266507131
hp_rno-mir-181d_st	4,539750658	2,857105825	2,342855193	3,246570559
hp_rno-mir-134_st	3,154259434	3,357467881	3,208447927	3,240058414
rno-miR-325-3p_st	2,726870373	4,774777485	2,143259687	3,214969182
hp_rno-mir-320_st	3,480306596	2,904353135	3,21403532	3,199565017
rno-miR-30b-3p_st	2,866669139	5,475296309	1,225700623	3,189222024
hp_rno-mir-503_st	2,932536993	3,671042124	2,911987122	3,171855413
hp_rno-mir-191_st	3,359258232	2,86619527	3,212932255	3,146128586
rno-miR-122_st	1,807860732	5,247801781	2,34566057	3,133774361
rno-miR-381_st	2,17334021	5,882516374	1,29174796	3,115868181
hp_rno-mir-361_st	2,993012178	3,291526487	2,993012178	3,092516947
rno-miR-27a-star_st	3,835646419	3,799322685	1,58811498	3,074361361
rno-miR-203_st	2,871202794	4,679245433	1,633099918	3,061182715
hp_rno-mir-92b_st	3,582724367	2,894984415	2,657531693	3,045080159
hp_rno-mir-23a_x_st	3,465655446	2,84261661	2,792031653	3,033434569
hp_rno-mir-383_st	2,712041567	3,167070823	3,167070823	3,015394405
hp_rno-mir-335_st	2,923661224	2,897162797	3,19791721	3,006247077
hp_rno-mir-26a_st	3,534974859	2,940492306	2,538703125	3,00472343
rno-miR-101a_st	1,817797387	4,70026084	2,443907788	2,987322005
hp_rno-mir-330_st	3,099567879	2,762039137	3,094646196	2,985417737
rno-miR-182_st	1,733563011	5,739045647	1,377070135	2,949892931
hp_rno-mir-375_st	2,940981605	2,921063717	2,904411761	2,922152361
rno-miR-872_st	2,681760427	4,692008453	1,371249687	2,915006189
hp_rno-mir-150_st	3,733463671	2,205727039	2,790031569	2,90974076
hp_rno-mir-874_st	3,501063844	2,648050514	2,5425864	2,897233586
hp_rno-mir-504_st	3,199969561	2,935271586	2,48991354	2,875051563
rno-miR-219-1-3p_st	2,751832095	3,401227717	2,46483379	2,872631201

rno-miR-147_st	2,698779481	1,091823078	4,810182214	2,866928258
hp_rno-mir-484_st	2,94802676	1,809261013	3,795193733	2,850827169
hp_rno-mir-140_st	2,595250316	2,961036069	2,951937041	2,836074475
hp_rno-mir-214_st	2,60424935	3,328738763	2,512217327	2,81506848
hp_rno-mir-127_st	2,733929456	2,927106668	2,658130532	2,773055552
hp_rno-mir-138-1_x_st	2,453652305	3,010137873	2,790081197	2,751290458
hp_rno-mir-500_st	3,098713991	2,881964635	2,21736232	2,732680315
hp_rno-mir-187_st	3,04794151	2,538570135	2,59436199	2,726957878
hp_rno-mir-181d_x_st	4,403984105	2,142270069	1,627961225	2,724738466
hp_rno-mir-195_st	2,757996907	2,578173519	2,814145274	2,7167719
hp_rno-mir-138-1_st	2,393337934	2,521767781	3,219506844	2,71153752
rno-miR-379-star_st	1,502906977	5,367206804	1,262697632	2,710937138
hp_rno-mir-93_st	2,507827871	2,883636015	2,73934483	2,710269572
hp_rno-mir-652_x_st	3,028361559	2,71524961	2,371014116	2,704875095
rno-miR-448_st	1,934596586	4,943873558	1,228565194	2,702345113
hp_rno-let-7a-1_st	2,648754483	3,141008946	2,297792764	2,695852065
hp_rno-mir-347_st	3,71631439	1,960040034	2,342443335	2,672932587
hp_rno-let-7b_st	2,533794416	2,880058993	2,604744158	2,672865856
rno-miR-133a_st	1,787192666	4,41840921	1,787192666	2,664264847
rno-miR-505_st	2,192458058	4,399794822	1,392451292	2,661568057
hp_rno-let-7d_st	2,90649796	2,478964066	2,553318176	2,646260067
rno-let-7i-star_st	2,019296009	4,536176175	1,371249687	2,642240624
hp_rno-mir-343_st	3,413139635	1,407456394	3,089597584	2,636731204
hp_rno-mir-328_st	2,409060688	1,941502881	3,536436366	2,628999978
rno-miR-675_st	5,131557981	1,287917435	1,454408514	2,624627976
hp_rno-mir-760_st	3,265487977	2,202680074	2,299413976	2,589194009
hp_rno-mir-678_st	3,933081626	1,679182989	2,136085612	2,582783409
hp_rno-mir-30c-2_st	2,621349675	2,821628925	2,293583415	2,578854005
rno-miR-223_st	2,230076789	2,392688327	3,108396025	2,577053714
rno-miR-186_st	1,912937895	4,37352976	1,398639447	2,561702367
rno-miR-760-5p_st	2,78917312	2,435330277	2,402256324	2,54225324
rno-miR-363_st	1,635632518	4,408937609	1,559330459	2,534633529
rno-miR-26b_st	1,903236464	4,122443669	1,441410126	2,489030086

rno-miR-101a-star_st	1,58167832	4,907133138	0,940392182	2,476401214
hp_rno-mir-193_st	2,541732753	2,224407803	2,658993394	2,47504465
rno-miR-19a_st	2,315271342	3,635353856	1,460655929	2,470427042
hp_rno-mir-673_st	1,971828375	2,078018841	3,339842937	2,463230051
rno-miR-296-star_st	2,988760866	2,410621875	1,981012224	2,460131655
rno-miR-374_st	2,128438761	3,85870916	1,371249687	2,452799203
hp_rno-mir-23a_st	2,743552972	2,161884099	2,439545483	2,448327518
rno-miR-501_st	2,82746323	3,649410253	0,86741606	2,448096514
hp_rno-mir-297_x_st	2,469492375	1,635632518	3,216233412	2,440452768
hp_rno-mir-299_x_st	2,21669459	2,531662311	2,552653009	2,43366997
hp_rno-mir-219-1_st	2,128438761	2,192458058	2,833340963	2,384745927
rno-miR-327_st	4,070622604	1,616128463	1,45556094	2,380770669
rno-miR-743b_st	1,72948245	2,332379823	3,051275443	2,371045905
rno-miR-382-star_st	1,665156964	4,273344081	1,170484793	2,369661946
hp_rno-mir-483_st	2,821347555	1,431536801	2,720720982	2,324535113
rno-miR-200c_st	2,157009098	3,245436578	1,524905382	2,30911702
rno-miR-136-star_st	1,198930824	4,52916136	1,198930824	2,30900767
hp_rno-mir-138-2_x_st	1,583376677	3,024724737	2,301786758	2,303296057
hp_rno-mir-143_st	2,359545354	1,894087974	2,654341181	2,30265817
hp_rno-mir-448_st	2,015884591	2,851366547	1,932668022	2,26663972
hp_rno-mir-412_st	1,809859806	2,814862161	2,163464386	2,262728784
hp_rno-mir-106b_st	2,339166433	2,316262267	2,067627829	2,241018843
hp_rno-mir-540_st	2,003576656	2,245695489	2,415187021	2,221486388
hp_rno-mir-338_st	2,104378934	2,743753943	1,81120444	2,219779106
hp_rno-mir-497_st	1,817214697	2,829454113	1,975608674	2,207425828
hp_rno-mir-674_st	2,128438761	2,25301952	2,193789405	2,191749229
rno-miR-20b-3p_st	2,192458058	2,312084991	2,050190005	2,184911018
hp_rno-mir-23b_st	2,468899584	1,791634893	2,285273817	2,181936098
hp_rno-mir-665_st	2,407170314	2,511950992	1,550221953	2,156447753
rno-miR-881_st	2,389946406	1,95929096	2,055672121	2,134969829
hp_rno-mir-212_st	2,245878203	2,332379823	1,81120444	2,129820822
hp_rno-mir-124-2_x_st	2,666069617	1,697870571	2,004318174	2,122752787
hp_rno-mir-675_st	2,651688129	1,893791025	1,81120444	2,118894531

rno-miR-99a-star_st	1,349695209	3,351993297	1,642475102	2,114721203
rno-miR-301b_st	1,019919661	3,904939529	1,371249687	2,098702959
rno-miR-24-1-star_st	1,444383358	3,458361283	1,366078493	2,089607712
hp_rno-mir-652_st	2,482696571	1,969196973	1,792784073	2,081559206
rno-miR-483_st	1,930037994	1,635632518	2,640066046	2,068578853
hp_rno-mir-598_st	1,903236464	1,802377995	2,492482879	2,066032446
hp_rno-mir-290_st	2,185574275	1,790810206	2,198855022	2,058413168
rno-miR-10a-5p_st	1,748491912	2,596868654	1,825361729	2,056907432
hp_rno-mir-666_st	2,50244977	1,702610418	1,898682404	2,034580864
rno-miR-133b_st	1,451917125	3,743507877	0,896419394	2,030614799
rno-miR-543_st	1,093989471	3,556619742	1,42994789	2,026852368
hp_rno-mir-132_st	1,783305411	2,297526986	1,992391873	2,02440809
hp_rno-mir-296_st	2,034511419	1,932061528	2,100069123	2,022214023
hp_rno-mir-221_st	1,73119293	2,192458058	2,134520342	2,019390443
hp_rno-mir-466c_st	2,015054872	1,272379338	2,760499331	2,015977847
hp_rno-mir-190_st	1,660203528	1,833455811	2,53338458	2,00901464
hp_rno-mir-30a_x_st	1,89176873	3,068148657	1,062488991	2,007468793
hp_rno-mir-128-1_st	1,859445507	2,224773316	1,927423844	2,003880889
rno-miR-33_st	0,537487835	4,155693934	1,296358895	1,996513555
hp_rno-mir-341_st	2,455775338	1,565420806	1,941031829	1,987409324
rno-miR-490_st	1,408368309	3,43733362	1,088229733	1,977977221
hp_rno-mir-210_st	2,412816941	1,80946008	1,703079473	1,975118831
rno-miR-484_st	2,008420499	2,242943854	1,667426634	1,972930329
rno-miR-184_st	1,443009701	3,624208427	0,840943097	1,969387075
hp_rno-mir-129-1_x_st	1,584944084	2,136305668	2,167446599	1,962898784
hp_rno-mir-487b_st	1,892563209	2,119272514	1,839892889	1,950576204
hp_rno-mir-342_st	1,689934329	2,320579467	1,802691547	1,937735114
hp_rno-mir-211_st	2,541570404	1,53539443	1,65620708	1,911057305
hp_rno-mir-188_st	1,959635644	1,669844469	2,048676372	1,892718828
hp_rno-mir-124-2_st	2,192458058	1,193498996	2,290124253	1,892027102
hp_rno-mir-125a_st	2,074223874	1,82296217	1,728404405	1,875196816
hp_rno-mir-877_st	2,453973759	1,620586882	1,507659036	1,860739892
rno-miR-499_st	1,82676897	1,920260873	1,81120444	1,852744761

hp_rno-mir-764_st	2,110220536	1,205646915	2,241032989	1,852300146
hp_rno-let-7b_x_st	1,858728762	1,770261637	1,923767213	1,850919204
hp_rno-mir-615_st	2,146752007	1,315723406	2,06841461	1,843630007
rno-miR-292-5p_st	2,168610273	1,796269241	1,559330459	1,841403324
hp_rno-mir-370_st	1,880111452	2,273308707	1,359894765	1,837771641
hp_rno-mir-28_st	1,908104573	2,158271089	1,434378736	1,833584799
rno-miR-711_st	2,279232763	1,704234875	1,464093902	1,815853847
hp_rno-mir-761_st	1,724755077	1,271601303	2,430976427	1,809110936
hp_rno-mir-758_x_st	1,326716025	1,590119231	2,494028938	1,803621398
hp_rno-mir-122_st	1,316709684	1,573494809	2,462605496	1,784269997
hp_rno-mir-708_x_st	1,550377797	2,352521463	1,438339518	1,780412926
hp_rno-mir-92a-2_st	2,821038453	0,950045723	1,568882266	1,779988814
hp_rno-mir-125b-1_x_st	2,033971087	1,472644669	1,772142021	1,759585926
hp_rno-mir-632_st	2,713374196	1,039483551	1,483429615	1,74542912
hp_rno-mir-301b_st	2,282505453	1,203296465	1,738821384	1,741541101
rno-miR-347_st	1,992198087	1,66904082	1,541656399	1,734298435
rno-miR-290_st	2,887321259	1,185610168	1,106557373	1,726496267
hp_rno-mir-671_st	1,862010029	1,810127155	1,502538086	1,724891757
hp_rno-mir-434_st	1,34776516	2,319870927	1,489050601	1,718895563
rno-miR-742_st	1,676639392	1,929628384	1,51947759	1,708581789
hp_rno-mir-205_st	1,704474161	1,707843296	1,711756388	1,708024615
hp_rno-mir-628_st	1,321847987	1,227634467	2,574401376	1,707961277
hp_rno-mir-668_st	1,269685172	1,988572174	1,863215068	1,707157471
hp_rno-mir-22_st	1,762926579	1,890311	1,445514477	1,699584018
hp_rno-mir-378_st	2,023906106	1,493523661	1,578548278	1,698659349
hp_rno-mir-152_st	2,128438761	1,68824098	1,276774014	1,697817918
hp_rno-mir-499_st	1,390091126	1,643492295	2,050471232	1,694684884
hp_rno-mir-194-2_st	2,40634545	1,449495876	1,217516708	1,691119345
hp_rno-mir-758_st	1,237168887	2,584336388	1,243941814	1,688482363
hp_rno-mir-466c_x_st	1,559330459	0,787662694	2,713374196	1,686789116
hp_rno-mir-34c_st	1,539835663	1,946561037	1,559330459	1,681909053
hp_rno-mir-9-1_x_st	1,383364697	2,143245191	1,502056284	1,676222057
hp_rno-let-7e_st	2,034582826	1,775278088	1,210135205	1,673332039

hp_rno-mir-129-2_x_st	1,611454799	1,271838966	2,115614663	1,666302809
rno-miR-21-star_st	1,953330596	1,456065818	1,576574051	1,661990155
hp_rno-mir-301a_st	1,450017764	1,635837517	1,889972517	1,658609266
hp_rno-mir-99a_st	1,4200794	2,04203631	1,50786637	1,656660693
hp_rno-mir-409_st	1,581638696	1,804280585	1,581638696	1,655852659
hp_rno-mir-128-2_st	1,302628885	1,730123064	1,917096525	1,649949492
hp_rno-mir-151_st	1,771617252	1,950101526	1,223307825	1,648342201
hp_rno-mir-421_st	1,67143188	2,29338879	0,979980263	1,648266978
hp_rno-mir-326_st	1,982958948	1,548589228	1,411491136	1,64767977
hp_rno-mir-145_st	1,747910311	1,914982219	1,271177034	1,644689854
hp_rno-mir-25_st	1,903236464	1,707872033	1,299536783	1,63688176
hp_rno-mir-376c_s_st	1,464280721	1,358675622	2,065054411	1,629336918
hp_rno-mir-455_st	2,093306803	1,652253607	1,118704016	1,621421476
hp_rno-mir-215_st	1,481086726	1,308379387	2,068632594	1,619366236
hp_rno-mir-125b-1_st	1,514195648	1,690785333	1,653062683	1,619347888
rno-miR-220_st	1,340961707	1,635632518	1,87235565	1,616316625
hp_rno-mir-9-1_st	1,544450175	2,149595229	1,154636537	1,616227314
hp_rno-mir-411_st	0,854234353	2,503269494	1,469593772	1,60903254
rno-miR-340-5p_st	0,945034218	2,732486884	1,148142574	1,608554558
hp_rno-mir-24-1_x_st	1,468982164	1,403978828	1,945021194	1,605994062
hp_rno-mir-218-2_st	1,50836434	1,628008428	1,660595031	1,598989267
hp_rno-mir-129-1_st	1,233008849	1,23151397	2,318063798	1,594195539
hp_rno-mir-297_st	1,357767305	0,610996481	2,799587439	1,589450408
hp_rno-mir-138-2_st	1,531530416	1,751556256	1,470525793	1,584537488
hp_rno-mir-130b_st	1,590283373	1,357724552	1,787342898	1,578450275
rno-miR-291a-5p_st	2,494924346	0,749552933	1,459322032	1,567933104
hp_rno-mir-200b_st	1,352182395	1,703226351	1,641931938	1,565780228
hp_rno-mir-128-2_x_st	0,985857015	1,83505496	1,870208546	1,56370684
hp_rno-mir-292_x_st	1,545591961	1,545591961	1,595996491	1,562393471
hp_rno-mir-490_st	1,193498996	1,334862191	2,132391023	1,55358407
hp_rno-mir-142_st	1,83505496	1,013675608	1,81120444	1,553311669
rno-miR-300-5p_st	0,84039546	2,932765906	0,872581062	1,548580809
hp_rno-mir-200c_st	1,69665237	1,498634109	1,440652068	1,545312849

hp_rno-mir-141_st	1,97247254	1,59615506	1,01130345	1,526643683
hp_rno-mir-551b_st	1,629767178	1,305430895	1,641389314	1,525529129
hp_rno-mir-431_st	1,122815433	1,743222097	1,703614861	1,523217464
hp_rno-mir-532_st	1,466225887	1,756844491	1,346305389	1,523125256
hp_rno-mir-384_st	1,249317301	2,17914601	1,126083441	1,518182251
hp_rno-mir-344-1_st	1,475018118	1,01632309	2,058396332	1,51657918
hp_rno-mir-30d_st	1,900773108	1,682186236	0,965790327	1,51624989
rno-miR-105_st	1,597481489	1,337790181	1,597481489	1,510917719
hp_rno-mir-30c-2_x_st	1,666185399	1,666185399	1,193732064	1,508700954
hp_rno-mir-192_st	1,802335126	1,286792548	1,436503663	1,508543779
hp_rno-mir-451_st	1,505681796	1,451797897	1,559330459	1,505603384
hp_rno-mir-182_st	1,34576165	1,715352209	1,425669843	1,495594567
hp_rno-mir-541_st	1,25219267	1,90727395	1,326266571	1,495244397
hp_rno-mir-194-2_x_st	1,489614327	1,480348386	1,51301364	1,494325451
rno-miR-664_st	1,579901801	1,635632518	1,262980321	1,492838213
hp_rno-mir-190_x_st	1,284562899	1,630662274	1,557147077	1,49079075
rno-miR-344-5p_st	1,573286453	1,275444115	1,616833578	1,488521382
rno-miR-292-3p_st	1,73119293	1,524006448	1,189784603	1,481661327
hp_rno-mir-147_st	1,445080592	1,337092947	1,591758265	1,457977268
rno-miR-183_st	0,830582657	2,584957131	0,955018445	1,456852744
hp_rno-mir-92a-2_x_st	1,775435678	1,337790181	1,254015602	1,455747153
hp_rno-mir-362_st	1,325185777	1,773010026	1,266015972	1,454737258
hp_rno-mir-101a_x_st	1,507977143	1,454837657	1,391868703	1,451561167
rno-miR-224_st	1,212796781	2,132789798	0,994312441	1,446633007
hp_rno-let-7a-2_st	1,537975716	1,294619344	1,503873169	1,445489409
hp_rno-mir-18a_st	1,375300579	1,413042984	1,540067568	1,442803711
hp_rno-mir-220-2_s_st	1,69891838	1,224886459	1,393957843	1,439254227
rno-miR-32_st	1,324955374	1,689833824	1,302701344	1,439163514
hp_rno-mir-377_st	1,249317301	1,83505496	1,226834342	1,437068867
hp_rno-mir-542_st	1,549653243	1,732282355	1,026611962	1,43618252
hp_rno-mir-376a_st	0,908244604	2,173746543	1,207171585	1,429720911
hp_rno-mir-345_st	1,662756201	1,301588885	1,313172614	1,425839233
rno-miR-592_st	0,981944986	2,36706101	0,92622731	1,425077769

rno-miR-340-3p_st	1,465158891	1,618762796	1,189662236	1,424527974
hp_rno-mir-379_st	0,910680063	2,035634247	1,32406171	1,423458673
rno-miR-376c_st	1,068524652	1,893794304	1,305325553	1,42254817
rno-miR-582_st	1,072067336	1,635632518	1,559330459	1,422343438
hp_rno-let-7a-1_x_st	1,431194883	1,435828815	1,388733776	1,418585825
hp_rno-mir-220-1_s_st	1,596392104	0,807521249	1,80671582	1,403543057
rno-miR-451_st	1,402966498	1,153107429	1,616879942	1,390984623
rno-miR-542-3p_st	1,697172436	1,451418693	1,019472797	1,389354642
rno-miR-878_st	1,344746916	1,647282443	1,175260296	1,389096552
hp_rno-mir-135b_st	1,393551031	1,196031865	1,577026237	1,388869711
hp_rno-mir-181c_st	1,711060095	1,508018032	0,945001131	1,388026419
hp_rno-mir-206_st	1,180598425	1,694393068	1,288453622	1,387815039
hp_rno-mir-495_st	1,433313565	1,640444875	1,059264265	1,377674235
rno-miR-1-star_st	1,416898701	1,635632518	1,072232241	1,374921153
hp_rno-mir-103-1_x_st	1,250430545	1,350809677	1,514858791	1,372033004
rno-miR-217_st	1,193498996	1,887245358	1,01709922	1,365947858
rno-miR-7b_st	0,674757724	2,369147147	1,053266605	1,365723825
hp_rno-mir-672_st	0,906071022	1,621230344	1,559330459	1,362210608
hp_rno-mir-27a_x_st	1,371249687	1,337790181	1,371249687	1,360096519
hp_rno-mir-153_st	1,301609348	1,254366392	1,522390334	1,359455358
hp_rno-let-7c-1_st	1,416627398	1,498634109	1,161756983	1,359006163
hp_rno-mir-224_st	1,83505496	1,483745106	0,747944759	1,355581608
hp_rno-mir-291a_st	1,686461475	1,281584224	1,07794402	1,34866324
v11_rno-miR-333_st	1,329960304	1,728084514	0,969029512	1,34235811
rno-miR-20a-star_st	1,197313816	1,310241662	1,506425509	1,337993662
hp_rno-mir-290_x_st	1,497467492	1,310152425	1,193498996	1,333706304
rno-miR-211_st	1,641278787	0,85804834	1,479745095	1,326357408
rno-miR-488_st	0,679136018	2,78917312	0,508041748	1,325450295
hp_rno-mir-322_st	1,249317301	1,424755949	1,302100979	1,325391409
hp_rno-mir-29a_st	1,170491046	1,313632457	1,491383149	1,325168884
hp_rno-mir-130a_st	1,257584771	1,730980814	0,986031194	1,324865593
hp_rno-mir-133a_st	1,25194042	1,498634109	1,216862712	1,32247908
hp_rno-mir-105_st	1,389866158	1,337790181	1,237206855	1,321621065

rno-miR-142-5p_st	1,324092391	1,261153974	1,371249687	1,318832018
hp_rno-mir-27a_st	1,64536176	1,110786061	1,193498996	1,316548939
hp_rno-mir-133b_st	0,958712548	1,205256644	1,783905797	1,31595833
hp_rno-mir-543_st	0,940975642	1,653801443	1,352873394	1,315883493
rno-miR-363-star_st	1,713848622	0,862217683	1,371249687	1,315771997
rno-miR-202_st	1,249317301	1,341560192	1,348555018	1,31314417
hp_rno-mir-881_st	1,131686032	1,381382319	1,418821817	1,310630056
hp_rno-mir-485_st	1,05335656	1,721101997	1,152573568	1,309010709
hp_rno-mir-27b_st	1,0482546	1,501685824	1,375720198	1,308553541
hp_rno-mir-871_x_st	1,249317301	1,256289672	1,418402803	1,308003259
hp_rno-mir-7a-2_st	1,411922387	1,027115283	1,475081155	1,304706275
hp_rno-mir-190b_x_st	1,413231705	1,385969619	1,080834507	1,293345277
hp_rno-mir-216a_st	0,961390614	1,516290407	1,385147486	1,287609503
hp_rno-mir-363_st	0,965460571	1,250252496	1,647046844	1,287586637
hp_rno-mir-376b_x_st	0,75756569	1,716434984	1,377030322	1,283676999
hp_rno-mir-181c_x_st	1,154788086	1,63503769	1,056578326	1,2821347
rno-miR-349_st	1,158688418	1,199196504	1,47984116	1,279242028
hp_rno-mir-494_st	0,908383245	1,499676579	1,423691674	1,277250499
hp_rno-mir-544_st	1,268315328	1,046619014	1,51668663	1,277206991
hp_rno-mir-30c-1_x_st	1,486584081	1,530029259	0,803831138	1,273481493
hp_rno-mir-199a_st	1,604902107	1,337790181	0,876577516	1,273089935
hp_rno-mir-410_st	1,054028787	1,648937055	1,11567075	1,272878864
hp_rno-mir-31_st	1,409417641	1,428184984	0,977670595	1,27175774
hp_rno-mir-222_st	1,172136348	1,592098727	1,035038256	1,266424444
rno-miR-200b_st	1,193498996	1,709435403	0,888427968	1,263787456
hp_rno-mir-379_x_st	0,87755131	1,668287398	1,241023311	1,26228734
hp_rno-mir-146b_st	1,443771828	1,301674545	1,038249531	1,261231968
hp_rno-let-7i_st	0,711810528	1,37736579	1,691996457	1,260390925
hp_rno-mir-196b_st	1,36071464	1,199587509	1,216515518	1,258939222
hp_rno-mir-124-1_x_st	0,663322273	2,054917521	1,04415444	1,254131412
hp_rno-mir-382_st	0,813133285	1,751205005	1,193498996	1,252612429
rno-miR-743a_st	1,249317301	1,464838006	1,03971582	1,251290375
hp_rno-mir-489_st	1,032525055	1,424863173	1,295995068	1,251127765

hp_rno-mir-124-3_x_st	1,144039298	1,685071587	0,922043529	1,250384805
rno-miR-30d-star_st	1,074373009	1,793710248	0,876577516	1,248220258
hp_rno-mir-33_st	1,166384766	1,337790181	1,232067258	1,245414068
hp_rno-mir-300_st	1,193498996	1,337790181	1,193498996	1,241596058
hp_rno-mir-26b_st	1,282609525	1,070458594	1,371249687	1,241439269
hp_rno-mir-201_st	1,193498996	1,193498996	1,321131971	1,236043321
hp_rno-mir-96_st	1,167509087	1,243394009	1,294533671	1,235145589
hp_rno-mir-429_st	1,243757029	0,989464588	1,471015094	1,23474557
hp_rno-mir-292_st	1,262385834	0,953866959	1,486513389	1,234255394
rno-miR-295_st	1,480638924	1,26644727	0,955269051	1,234118415
hp_rno-mir-568_st	1,102419324	1,151364894	1,447915841	1,23390002
hp_rno-mir-327_st	1,275511484	1,258306741	1,16752384	1,233780688
hp_rno-mir-369_st	1,173964376	1,345550192	1,168182853	1,229232473
hp_rno-mir-34b_st	1,299013991	1,193498996	1,187405528	1,226639505
hp_rno-mir-98_st	1,069691508	1,62792316	0,97791951	1,225178059
hp_rno-mir-496_st	0,862857498	1,695949781	1,113447265	1,224084848
hp_rno-mir-24-2_st	1,524219075	1,157406502	0,97474349	1,218789689
hp_rno-mir-582_st	0,72536669	1,474447866	1,453848813	1,21788779
hp_rno-mir-331_st	1,186167596	1,165474508	1,292499091	1,214713732
hp_rno-mir-340_st	1,125505261	1,38021781	1,133673714	1,213132261
hp_rno-let-7c-2_x_st	0,781172195	1,013675608	1,837032177	1,21062666
hp_rno-mir-125b-2_st	1,03716701	1,413201329	1,175723462	1,208697267
hp_rno-mir-146a_st	0,925610024	1,204851874	1,494976692	1,20847953
hp_rno-mir-101a_st	0,922439402	1,336289002	1,366385466	1,20837129
hp_rno-mir-743a_st	1,57387522	0,857540577	1,193498996	1,208304931
hp_rno-mir-30e_x_st	1,193498996	1,194078044	1,213114983	1,200230674
hp_rno-mir-29b-2_x_st	1,336533752	1,572210454	0,686130868	1,198291692
hp_rno-mir-298_st	0,994190362	1,406917282	1,193498996	1,198202213
rno-miR-489_st	1,125155115	1,18106432	1,284398247	1,196872561
hp_rno-mir-1_st	1,069691508	1,343795908	1,171166346	1,194884587
hp_rno-mir-20b_st	1,339586666	0,878986959	1,364343462	1,194305696
hp_rno-mir-30a_st	1,337894409	1,214090606	1,026611962	1,192865659
hp_rno-mir-879_st	1,013295725	1,191519864	1,370136701	1,191650763

hp_rno-mir-1224_st	1,306248515	1,193498996	1,075043589	1,191597033
hp_rno-mir-466b-1_x_st	1,376480173	0,966779994	1,229968507	1,191076225
hp_rno-mir-29c_st	1,153212534	1,389288189	1,026611962	1,189704228
hp_rno-mir-871_st	1,343105202	1,24400228	0,979879605	1,188995695
hp_rno-mir-100_st	1,278438532	1,258907989	1,026611962	1,187986161
hp_rno-mir-352_st	0,951449255	1,410661655	1,198298423	1,186803111
hp_rno-mir-294_st	1,459428855	0,918090605	1,175863604	1,184461021
hp_rno-mir-218-1_st	1,22392283	1,22392283	1,105182912	1,184342857
hp_rno-mir-295-1_s_st	1,478562754	1,422289701	0,645175876	1,182009444
rno-miR-471_st	0,857540577	1,649830108	1,03165601	1,179675565
hp_rno-mir-219-2_st	1,039698442	1,318125943	1,181027851	1,179617412
hp_rno-mir-181a-1_st	1,069288279	1,446516102	1,013469974	1,176424785
rno-miR-376b-5p_st	1,111471285	1,142828655	1,265953603	1,173417848
hp_rno-mir-501_st	1,006250152	1,356196827	1,157487851	1,17331161
hp_rno-mir-128-1_x_st	1,158123869	1,208323271	1,150215853	1,172220998
hp_rno-mir-200a_st	1,210958609	1,098838875	1,203077461	1,170958315
hp_rno-mir-103-2_x_st	0,847829971	1,480510422	1,164354354	1,164231582
hp_rno-mir-9-2_x_st	0,874887901	1,59827072	1,019137785	1,164098802
hp_rno-mir-511_st	1,018800851	1,415822043	1,053004634	1,162542509
hp_rno-mir-664-2_x_st	1,080325934	1,498634109	0,907742547	1,162234197
rno-miR-201_st	1,263318386	1,127304551	1,094343643	1,161655526
hp_rno-mir-10b_st	1,202467861	0,938345186	1,343988391	1,161600479
hp_rno-mir-30b_st	0,880891791	1,46734551	1,107873567	1,152036956
hp_rno-let-7f-1_st	0,965000302	1,385697493	1,103556754	1,151418183
hp_rno-mir-181b-2_st	1,339093089	1,007395486	1,106696817	1,151061797
hp_rno-mir-183_st	1,387973324	0,782405401	1,275416207	1,148598311
rno-miR-294_st	1,319675807	1,051924117	1,070961056	1,147520327
hp_rno-mir-872_st	0,90286846	1,517394008	1,021899611	1,14738736
hp_rno-mir-708_st	0,966462247	1,26817494	1,193498996	1,142712061
hp_rno-let-7c-2_st	0,743480964	1,353877798	1,325534803	1,140964522
rno-miR-336_st	0,854992998	1,195759689	1,3649178	1,138556829
hp_rno-mir-743a_x_st	1,371249687	1,013675608	1,012726677	1,132550657
hp_rno-mir-349_st	0,910680063	1,657946636	0,823170038	1,130598912

hp_rno-mir-21_st	1,020608028	1,430564141	0,926759591	1,125977253
hp_rno-mir-449a_st	0,961371464	1,193498996	1,210475001	1,12178182
hp_rno-mir-493_st	1,264613267	0,879088482	1,220856311	1,121519353
rno-miR-429_st	1,287682874	0,918989359	1,150584782	1,119085672
hp_rno-mir-204_st	0,958860648	1,168098797	1,224441683	1,117133709
rno-miR-10a-3p_st	0,893664437	1,194890974	1,25958681	1,116047407
hp_rno-mir-433_st	1,013675608	1,337790181	0,99283956	1,11476845
hp_rno-mir-742_st	1,149367898	1,164380016	1,02543548	1,113061132
hp_rno-mir-101b_st	0,749552933	1,023210374	1,559330459	1,110697922
rno-miR-293_st	0,749552933	1,115576997	1,466696164	1,110608698
rno-miR-126-star_st	0,883372628	1,237801165	1,21012583	1,110433208
hp_rno-mir-203_st	1,262378553	1,013675608	1,047230326	1,107761496
hp_rno-let-7f-1_x_st	1,065644968	1,34901101	0,897521088	1,104059022
hp_rno-mir-495_x_st	0,764773415	1,295092959	1,245580761	1,101815712
rno-miR-632_st	1,117294093	1,083708384	1,092346123	1,097782867
hp_rno-mir-329_st	1,083181292	1,169008728	1,039818569	1,097336196
rno-miR-365_st	0,868023284	1,313375716	1,104826911	1,095408637
rno-miR-141_st	0,836294468	1,124148093	1,32367052	1,094704361
hp_rno-mir-488_st	1,127448015	1,193498996	0,959545616	1,093497542
rno-miR-219-2-3p_st	0,952115483	1,19865958	1,120507418	1,090427494
hp_rno-mir-20a_st	1,144062488	1,033241027	1,089451785	1,088918433
hp_rno-mir-376c_st	0,906365481	0,964850704	1,38805395	1,086423378
hp_rno-mir-365_st	0,95765586	1,025519787	1,270063156	1,084412934
rno-miR-26b-star_st	1,326855551	1,040254952	0,882909487	1,083339997
hp_rno-mir-129-2_st	1,193498996	1,114289059	0,929303754	1,079030603
hp_rno-mir-711_st	1,213759827	1,073838062	0,948465701	1,078687863
hp_rno-mir-136_st	0,803546755	1,159291488	1,270771205	1,077869816
hp_rno-mir-208_st	1,052513994	1,252001563	0,92264112	1,075718893
hp_rno-mir-32_st	1,10366341	1,227470897	0,893563467	1,074899258
hp_rno-mir-664-1_x_st	0,967617086	1,444966713	0,806050641	1,072878147
hp_rno-mir-301b_x_st	0,984119757	1,072592637	1,147485915	1,068066103
hp_rno-mir-299_st	0,850849616	1,361866754	0,980811197	1,064509189
rno-miR-759_st	0,916520429	1,04630043	1,226123818	1,062981559

hp_rno-mir-29b-1_x_st	0,907903903	1,356680156	0,920840256	1,061808105
hp_rno-mir-30e_st	0,974692706	1,042566843	1,167200678	1,061486743
hp_rno-mir-380_st	0,696764894	1,742898864	0,742121425	1,060595061
hp_rno-mir-471_st	0,826829861	1,378183793	0,973165895	1,059393183
hp_rno-mir-505_st	0,578511144	1,609752494	0,984383204	1,057548947
hp_rno-let-7f-2_x_st	1,292405871	0,857540577	1,019990762	1,056645736
rno-miR-196c_st	0,763216601	1,404310133	0,990433546	1,052653427
hp_rno-mir-7a-1_st	1,024820438	1,204247435	0,923615654	1,050894509
rno-miR-196a-star_st	1,371249687	0,841368712	0,931337962	1,047985454
hp_rno-mir-323_st	0,78620006	1,301807034	1,053862754	1,047289949
hp_rno-let-7f-2_st	1,013675608	0,930965245	1,193498996	1,046046616
hp_rno-mir-196c_st	0,933057271	0,698121932	1,497650233	1,042943145
hp_rno-mir-154_st	1,055425102	1,037065833	1,026611962	1,039700966
hp_rno-mir-336_st	0,819829608	0,84875026	1,448756526	1,039112131
rno-miR-215_st	0,858755045	1,064131173	1,193498996	1,038795071
hp_rno-mir-126_st	0,687464422	1,265644588	1,158355864	1,037154958
hp_rno-mir-376b_s_st	1,071924686	0,853538453	1,179671234	1,035044791
rno-miR-513_st	0,702383706	1,24554032	1,150061817	1,032661948
rno-miR-653_st	0,815516774	1,348819254	0,928752042	1,031029357
hp_rno-mir-450a_st	1,154235821	0,81842568	1,117558112	1,030073204
hp_rno-mir-466b-2_x_st	0,952620658	0,845892661	1,289838724	1,029450681
hp_rno-mir-190b_st	0,884844372	1,027774866	1,170652169	1,027757136
hp_rno-mir-293_st	0,866123391	1,337790181	0,876577516	1,026830363
hp_rno-mir-295-2_s_st	1,178051863	0,803546755	1,080605784	1,0207348
rno-miR-208_st	1,105866595	0,944739464	0,999700097	1,016768719
hp_rno-mir-29b-2_st	0,802961065	1,635632518	0,608007013	1,015533532
rno-miR-216a_st	1,290386407	1,095553576	0,652065586	1,012668523
rno-miR-369-3p_st	0,90579566	1,006272674	1,110821242	1,007629859
hp_rno-mir-294_x_st	0,99007226	0,729801051	1,297879719	1,005917677
hp_rno-mir-92a-1_st	0,835370085	1,304940378	0,87311249	1,004474318
rno-miR-200a_st	1,079998682	0,89509061	1,03666316	1,003917484
hp_rno-mir-223_st	0,837375324	1,018457341	1,145481924	1,000438196
rno-miR-1_st	0,749552933	1,247524429	0,99898005	0,998685804

rno-miR-628_st	1,2053167	1,274856685	0,511840128	0,997337838
hp_rno-mir-7b_st	0,718542783	1,09972559	1,158295583	0,992187985
hp_rno-mir-802_st	1,069691508	1,028478704	0,876577516	0,991582576
hp_rno-mir-513_st	1,047018701	0,942323769	0,979312904	0,989551792
hp_rno-mir-181a-2_st	1,058462914	0,903265914	1,005981617	0,989236815
hp_rno-mir-9-2_st	0,851075969	1,138709746	0,975516456	0,988434057
hp_rno-mir-883_st	0,771874458	0,78231371	1,396763778	0,983650648
rno-miR-10b_st	1,010524165	0,879553154	1,026611962	0,97222976
rno-miR-196b_st	0,583679728	1,265644588	1,065368197	0,971564171
rno-miR-875_st	0,749552933	1,316856722	0,845024043	0,970477899
hp_rno-mir-103-2_st	1,022743299	1,013675608	0,87298097	0,969799959
hp_rno-mir-137_st	0,909495901	1,173618576	0,824038823	0,9690511
rno-miR-879_st	0,887401955	1,005023441	1,014426539	0,968950645
rno-miR-29b-1-star_st	0,906742269	0,933841907	1,030549756	0,957044644
hp_rno-mir-29b-1_st	0,884411171	1,177331233	0,806407475	0,95604996
hp_rno-mir-743b_st	1,006881838	0,829131146	1,026611962	0,954208315
hp_rno-mir-181a-1_x_st	1,033156542	1,019918046	0,804470823	0,952515137
hp_rno-mir-184_st	0,833450789	1,022030724	0,998217778	0,951233097
hp_rno-mir-547_st	1,140863038	0,857540577	0,84963447	0,949346028
hp_rno-mir-300_x_st	0,744950138	1,091350049	0,997185559	0,944495248
rno-miR-871_st	0,749552933	1,097884878	0,984957032	0,944131614
hp_rno-mir-148b_st	0,958814167	1,097370619	0,773377631	0,943187472
rno-miR-144_st	0,753623238	0,89217969	1,169238719	0,938347216
rno-miR-511_st	0,837189151	1,013076023	0,950162834	0,933476003
hp_rno-mir-876_st	0,98666974	0,92549144	0,877717551	0,929959577
hp_rno-mir-376b_st	0,863088798	1,262897973	0,653877416	0,926621396
hp_rno-mir-202_st	0,962177836	0,888779185	0,92642889	0,925795303
hp_rno-mir-125b-2_x_st	0,749552933	1,183006523	0,838192139	0,923583865
hp_rno-mir-181b-1_st	0,938063242	1,082354426	0,748773911	0,92306386
hp_rno-mir-16_st	0,937179963	1,043945601	0,786439352	0,922521639
rno-miR-376a-star_st	0,883574884	1,007511016	0,849472337	0,913519412
hp_rno-mir-10a_st	0,849984041	0,852164575	1,031987963	0,91137886
hp_rno-mir-653_st	0,674673824	1,235637328	0,822585589	0,91096558

hp_rno-mir-381_st	0,836144389	1,289176249	0,593745888	0,906355509
hp_rno-mir-592_st	0,857540577	0,887738188	0,953938539	0,899739101
hp_rno-mir-194-1_st	0,783795565	0,995814438	0,919217274	0,899609092
hp_rno-mir-325_st	0,749279502	0,974386596	0,974386596	0,899350898
hp_rno-mir-873_st	0,884034213	1,086384815	0,721536653	0,89731856
hp_rno-mir-875_st	0,836625523	0,917442784	0,937196157	0,897088155
hp_rno-mir-19a_st	0,711810528	1,066951095	0,909332386	0,896031336
hp_rno-mir-144_st	0,750080852	1,157424329	0,779478897	0,895661359
hp_rno-mir-465_st	0,848253916	1,094798013	0,742178451	0,895076793
hp_rno-mir-30c-1_st	0,692952054	1,377794676	0,611355544	0,894034091
hp_rno-mir-880_st	1,104469265	0,86675156	0,70389512	0,891705315
hp_rno-mir-19b-1_st	0,541292113	1,235398609	0,881754497	0,886148406
rno-miR-219-5p_st	0,857540577	0,83461744	0,963981163	0,885379727
hp_rno-mir-124-1_st	0,582247163	0,917725616	1,147088541	0,882353773
hp_rno-mir-878_st	0,964743082	1,013675608	0,657808395	0,878742362
hp_rno-mir-350_st	0,891077719	0,696283952	1,045718661	0,877693444
hp_rno-mir-539_st	0,610310072	1,137034127	0,876577516	0,874640572
hp_rno-mir-19b-2_st	0,647058657	0,813446199	1,162315379	0,874273412
hp_rno-mir-92a-1_x_st	0,839053135	0,749552933	1,026611962	0,871739343
hp_rno-mir-376c_x_st	0,710403856	1,193498996	0,694952887	0,866285246
hp_rno-mir-135a_st	0,867059047	0,857540577	0,867059047	0,863886223
rno-miR-190b_st	0,624433829	1,040004217	0,9262645	0,863567515
hp_rno-mir-759_st	0,800934896	1,015016763	0,773592493	0,863181384
rno-miR-568_st	0,516542527	1,023380512	1,045085854	0,861669631
hp_rno-mir-196a_st	0,80199202	0,864527614	0,907869073	0,858129569
hp_rno-mir-17-2_st	0,949934495	0,949934495	0,663535784	0,854468258
rno-miR-883_st	0,694669994	1,138616057	0,720615338	0,851300463
rno-miR-463_st	0,523567816	1,132135987	0,89447273	0,850058844
hp_rno-mir-34a_st	0,872889853	1,029024884	0,61242764	0,838114126
rno-miR-148b-5p_st	0,756792188	0,68611276	1,046592746	0,829832565
hp_rno-mir-217_st	0,698947968	0,915215406	0,840351148	0,818171507
rno-miR-135b_st	0,717071946	0,963616043	0,772517498	0,817735162
hp_rno-mir-186_st	0,763525715	0,878245656	0,782122776	0,807964716

rno-miR-190_st	0,606826952	0,674345785	1,139449961	0,806874233
rno-miR-196a_st	0,843367401	0,593704956	0,959536419	0,798869592
rno-miR-880_st	0,819342311	0,946379439	0,586907497	0,784209749
hp_rno-mir-24-1_st	0,699086618	0,652743146	0,98190555	0,777911771
hp_rno-mir-463_st	0,909712998	0,910992317	0,509162774	0,776622696
rno-miR-29a-star_st	0,571717779	0,9302	0,826210798	0,776042859
hp_rno-mir-19b-1_x_st	0,679054879	0,713902517	0,909333165	0,767430187
rno-miR-421_st	0,739624805	1,010195132	0,546510813	0,765443584
hp_rno-mir-103-1_st	0,809171156	0,857540577	0,619738951	0,762150228
hp_rno-mir-107_st	0,616218346	0,913516903	0,75560664	0,76178063
hp_rno-mir-17-2_x_st	0,719763202	1,019695252	0,54363231	0,761030255
rno-miR-802_st	0,906324993	0,530417173	0,840598989	0,759113718
rno-miR-761_st	0,84045942	0,653082075	0,782445799	0,758662431
rno-miR-142-3p_st	0,463738414	0,785441777	1,026682816	0,758621002
rno-miR-384-3p_st	0,693360923	0,81805016	0,749552933	0,753654672
hp_rno-mir-15b_st	0,397675115	0,945045528	0,918021415	0,753580686
rno-miR-136_st	0,498068635	0,869041433	0,869041433	0,745383834
hp_rno-mir-374_st	0,612054122	0,780263268	0,776576027	0,722964472
rno-miR-876_st	0,791685895	0,625006408	0,722563528	0,713085277
rno-miR-450a_st	0,703568039	0,750671203	0,6406931	0,698310781
rno-miR-544_st	0,58768979	0,882944053	0,606726729	0,692453524
rno-miR-135a_st	0,495730003	0,692961458	0,654907559	0,614533007
rno-miR-547_st	0,475770564	0,710705903	0,610996481	0,599157649
rno-miR-96_st	0,682107592	0,343741825	0,763645058	0,596498158

al neural cells.
